# Recent advances in nanomedicine preparative methods and their therapeutic potential for colorectal cancer: a critical review

**DOI:** 10.3389/fonc.2023.1211603

**Published:** 2023-06-22

**Authors:** Arinjay Jain, Sankha Bhattacharya

**Affiliations:** Department of Pharmaceutics, School of Pharmacy & Technology Management Shirpur, SVKM’S NMIMS Deemed-to-be University, Shirpur, Maharashtra, India

**Keywords:** colorectal cancer, nanomaterials, anticancer drug, tumor microenvironment, enhanced permeability and retention

## Abstract

Colorectal cancer (CRC) is a prevalent malignancy that affects a large percentage of the global population. The conventional treatments for CRC have a number of limitations. Nanoparticles have emerged as a promising cancer treatment method due to their ability to directly target cancer cells and regulate drug release, thereby enhancing therapeutic efficacy and minimizing side effects. This compilation examines the use of nanoparticles as drug delivery systems for CRC treatment. Different nanomaterials can be used to administer anticancer drugs, including polymeric nanoparticles, gold nanoparticles, liposomes, and solid lipid nanoparticles. In addition, we discuss recent developments in nanoparticle preparation techniques, such as solvent evaporation, salting-out, ion gelation, and nanoprecipitation. These methods have demonstrated high efficacy in penetrating epithelial cells, a prerequisite for effective drug delivery. This article focuses on the various targeting mechanisms utilized by CRC-targeted nanoparticles and their recent advancements in this field. In addition, the review offers descriptive information regarding numerous nano-preparative procedures for colorectal cancer treatments. We also discuss the outlook for innovative therapeutic techniques in the management of CRC, including the potential application of nanoparticles for targeted drug delivery. The review concludes with a discussion of current nanotechnology patents and clinical studies used to target and diagnose CRC. The results of this investigation suggest that nanoparticles have great potential as a method of drug delivery for the treatment of colorectal cancer.

## Graphical Abstract

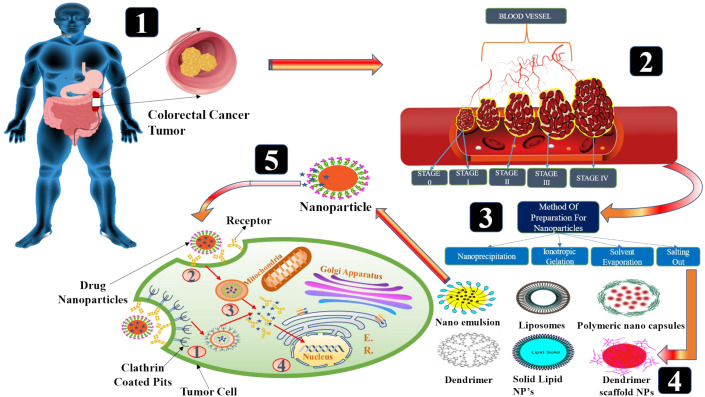

## Introduction

In many cases, a cancerous growth called colorectal cancer (CRC) can be found in the gastrointestinal tract. Currently, CRC is the third leading cause of death in the globe (1). Around a quarter of all cancer cases are due to CRC, making it one of the most common malignancies. Younger patients are increasingly more likely to have colon cancer (2). Economic and living conditions have steadily improved over the past few decades, which has led to an increase in CRC (3). Colorectal cancer (CRC) can be caused by genetic mutations, just like other types of cancer. These mutations affect oncogenes, tumor suppressors, and genes involved in DNA repair processes (4). Depending on the source of the mutation, CRC can be classified as sporadic, hereditary, or familial. Sporadic CRC refers to cancers that occur during a person’s lifetime and are caused by mutations that are not related to genetic diseases. These mutations affect people’s brains and their offspring and account for 70 percent of all colon cancers. The molecular mechanisms behind cancer are diverse and target many genes (5). However, approximately 70% of CRC patients follow a specific mutation pattern that results in characteristic mutations. The system usually starts with a mutation in the adenomatous polyposis coli (APC) gene, a tumor that causes benign adenoma or polyp formation. Subsequent mutations in genes such as KRAS, TP53 and DCC contribute to the disease. Inherited cancers represent a small fraction, comprising only 5% of all colorectal cancer cases. These cancers arise from inherited mutations affecting one allele of the mutated gene. When a point mutation occurs in the other allele, it initiates the formation of tumor cells and eventually carcinoma development (6, 7). On the other hand, familial CRC comprises around 25% of all cases and is also caused by inherited mutations. However, it is not classified as an inherited cancer in the strict sense, as it does not fit into any specific inherited cancer syndrome variant (8).

In addition to genetic mutations, various personal traits and habits are recognized as risk factors for the development of CRC or polyps. Advancing age is a significant risk factor, with the chances of developing CRC notably increasing after the age of fifty, while occurrences before this age are rare except in cases of inherited cancers (9). Recent research indicates that individuals diagnosed with bowel diseases tend to have a heightened awareness of colorectal cancer compared to others (10–14). Conditions such as inflammatory bowel disease, chronic ulcerative colitis, and Crohn’s disease, particularly when accompanied by adenomatous polyps, are considered primary lesions that elevate the risk of developing CRC (15–18). Family history also plays a significant role, as numerous studies have demonstrated a 2.5 to 3 times greater risk of colorectal cancer among relatives of affected individuals (19).

Other risk factors, such as leading a sedentary lifestyle, can elevate the likelihood of developing colorectal cancer (20). A sedentary lifestyle is often associated with obesity, another major risk factor for colon cancer. More importantly, this increased risk has been attributed to dietary choices and the accumulation of adipose tissue (VAT), the metabolic equivalent of total body fat. VAT promotes colon cancer development by causing inflammation in the colon and rectum by releasing proinflammatory cytokines. This process also causes insulin resistance and affects metabolic enzymes such as adiponectin and lectins (21). Smoking and alcohol increase the risk of colon cancer. Alcohol, especially its metabolite acetaldehyde, is considered carcinogenic and increases the risk of cancer, especially in individuals with specific enzymes that metabolize alcohol. Smoking, on the other hand, was associated with a 10.8 percent increased risk of lung cancer, mainly due to the presence of carcinogens such as nicotine, which can reach the stomach and cause polyps (22).

CRC is cured with a variety of surgical, radiation, chemotherapy, and other modalities, including immunotherapy and targeted therapy (23). Because CRC is difficult to detect in its earliest stages, people who present with symptoms are almost always in the middle or later stages of the illness. Drug resistance and recurrence of CRC may result from the presence of tumor stem cells (24). Recent advances in pharmaceutical colloidal system preparation have made it possible to develop drug carriers that are both safe and effective. Liposomes, niosomes, polymeric, nanoparticles, micelles, gold nanoparticles, and other colloidal carriers are examples of drug delivery systems. NPs have risen to the top for the drug delivery due to an increase in their therapeutic efficacy over the last decade. NPs are solid colloidal particles with a diameter ranging from 10 nm to 1000 nm used in pharmaceuticals (25). There are three ways to deliver medication or biologically active ingredient: dissolving, encapsulating, or attaching to the surface of macromolecular molecules. To make nanoparticles of one type or the other, a variety of different preparation methods and starting materials are required. The morphology and structure of these two kinds are vastly different. A dense polymeric matrix makes up nanospheres, whereas a polymeric membrane encloses the core of nano capsules (1).. By acting as a unique carrier for biomacromolecules, nanoparticles can enhance ingestion and absorption of insoluble medications and targeted release pharmaceuticals, as well as achieve precisely focused therapy (26).. Antitumor medications can be delivered to specific tumours using this method in a variety of ways, including passive and active targeting (27). Passive targeting indicates that the nano DDS can efficiently accumulate in the tumor depending on the physiological and pathological properties of the tumor location and the nature of the nano delivery system (23). Tumours have a distinct microenvironment compared to healthy tissue. Because the microvascular structure of solid tumours differs from that of normal tissue, macromolecules and massive particles are unable to permeate the capillary wall because the endothelial space is dense and complete. Solid tumor tissue has many new blood vessels, the vascular wall space is broad, the structural integrity is poor, and lymphatic reflux is absent (28). Macromolecular drugs or particles with diameters of 100 nm are more likely to accumulate in tumor tissue because of this difference; additionally, specific pH, enzyme environment, and reduction environment in tumor site can be used to achieve the release of drugs at specific sites in order to achieve the goal of targeted drug delivery (29). Tumor cells proliferate rapidly, resulting in a lack of blood vessels and lymphatics in the tumor tissue, which results in a high rate of leakage of substances from blood vessels into the tumor tissue, which can’t easily return to the lymphatic vessels, increasing the retention and infiltration of the tumor (30). To distribute macromolecules or nanoparticles by tumor extravasation, the retention effect of solid tumours is unique. Tumor vascular endothelium has huge gaps that allow macromolecule medicines to selectively extravasate into tumor tissue as a result of increased vascular density brought on by angiogenesis in solid tumours (31). For example, EPR-based cancer therapy for macromolecular cancers could be used to treat more tumours. Nitroglycerin has been shown to boost the EPR impact of tumours by increasing the transfer of medications to tumours by 2-3-fold and thereby improving the therapeutic effect (32). Deficiencies in tumor lymphatics can potentially increase tumor interstitial pressure and hinder the diffusion of medicines within the tumor. Nanocarriers modified by hydrophilic polymer materials serve as active targeting agents, delivering medications to specific organs or tissues (33). For example, in contrast to passive drug targeting, active targeting is the combination of active recognition between specific molecules on the surface of the nanosystem and specific molecules and proteins on the tumor site in order to obtain selective drug concentrations in tumor tissue and cells. Most active drug targeting is aimed at improving target cell identification and uptake rather than increasing total tumor accumulation (34). Targeted nano-drug preparations can be made by combining polymer nano-carriers with precise combinations of tumor cell surface receptors or antigens, allowing for the active delivery of medicines. Pharmacological nanocarriers that can remain in the bloodstream for a long period of time—such as liposomes, micelles, or polymeric nanoparticles—may be utilized to transport drugs into tumours by passive accumulation. It is common for nano drug carriers to have a long *in-vivo* half-life (35). Targeting tumor cells without damaging non-tumor cells is now possible due to improvements in targeted drug delivery. Different nanoparticles are being formulated and investigated for the efficient transport of cytotoxic drugs to the target site, improving drug distribution and bioavailability while concurrently reducing adverse effects. Immunotherapy still remains only an experimental approach despite the fact that few clinical trials have shown the ability to help patients with CRC. For successful CRC treatment, evaluation of the ongoing and finished studies is required. Researchers are working to create new carrier systems that might improve the targeting capacity of chemo- and immune-therapeutics with poor therapeutic index. Numerous preclinical investigations have shown that nanotherapy is more effective than traditional methods in treating CRC (36).

So that medications have a longer half-life and are more readily absorbed by tumor tissue due to their hydrophilic groups, nano-drug carriers can inhibit macrophage system affinity. The lifespan and quality of life of cancer patients, particularly in the late stages of the disease, have been dramatically diminished as a result of multidrug resistance and harm to normal cells. Some 20 to 400 nanometer-sized drug-loaded nanoparticles such as liposomes and dendrimers, as well as micelles, have improved drug delivery for CRC therapy in recent years (37). Nanoparticle-based drug delivery systems can increase medication bioavailability, reduce adverse effects, and protect healthy cells by delivering pharmaceuticals to the target spot (38). Small-molecule antitumor drugs, genes, or proteins can be transported by nanocarriers, which can avoid normal tissues while allowing the drugs to accumulate in tumor tissue, thus increasing their concentration in tumours while reducing the toxicity of the remaining body compared to the effects observed with free drugs (39). To top it all off, nanopillars provide a number of advantages over conventional pharmaceuticals, including lower renal clearance and better drug half-lives, controlled release and improved solubility. Despite the rapid development of nanomaterials, few nano agents have been successfully employed for tumor therapy at the present time (40).

## Colorectal cancer stages

**Figure 1:**
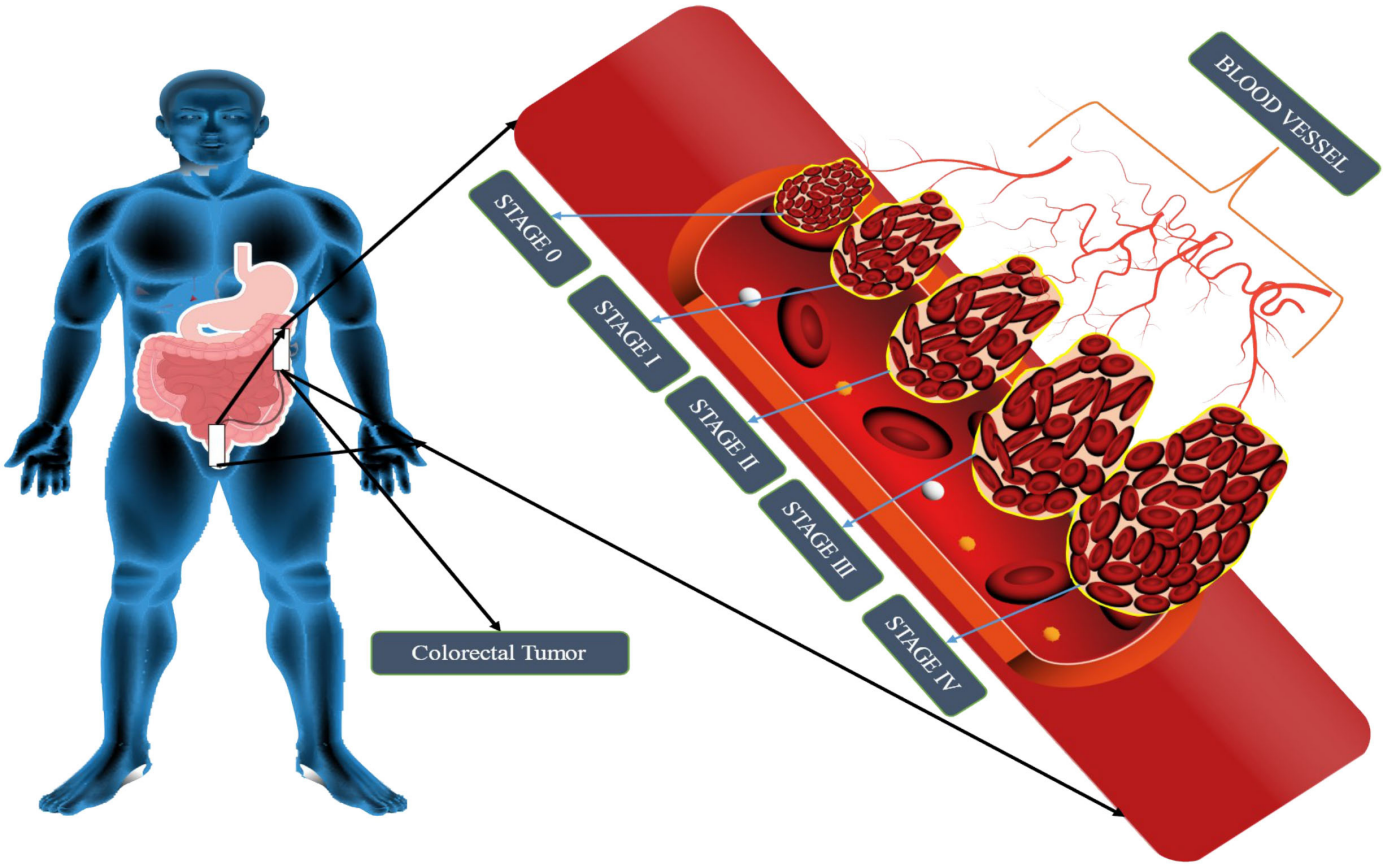
Schematic representation of CRC stages. CRC tumor growth progresses via four stages: metastasis, progression, promotion, and initiation. The liver, lung, and bone are the most typical metastatic sites. It is impossible to estimate how long each stage will take, but decades will probably be needed to develop CRC.

Staging is the process of determining whether or not cancer has migrated from the colon/rectum to other areas of the body (41). Staging is important because it helps decide the best course of action for therapy. Oncologists use the term “stage” to describe how far cancer has spread. Treatment options for colon cancer must be determined based on its stage. The TNM staging system, developed by the Union for International Cancer Control and endorsed by the American Joint Committee on Cancer, is used to classify colon cancer. For patients diagnosed with metastatic CRC, the survival rate ranges from 90 percent to 10 percent, depending on the disease stage (42). The greater the possibility of survival, the earlier in the disease process the diagnosis is made. Cancer’s level in the body is one of the most important criteria in determining which therapy will be most effective and how successful it will be. Colorectal cancer is depicted in **Figure 1**. When abnormal cells form in the colonic mucosa, they have the potential to develop into malignancies (43). In stage I, cancer has spread from the mucosa of the colon wall into the submucosa and the muscularis propria of the colon. After spreading to the visceral peritoneum (IIB) and the connected organs, cancer in stage II spreads farther from the muscularis propria into the peri-colorectal tissues (IIA) (IIC) (**Figure 1**). Muscularis propria metastases in surrounding tissues or 1–3 regional lymph nodes or submucosa spreading with metastases in 4–6 lymph nodes and IIIB and 7 or more regional lymph nodes in stage (IIIC) (44). Stage IV cancer is further subdivided into IV A and IV B, with metastatic spread confined to a single organ s3uch as the liver, ovary, lung, regional node, etc. (45). A 5 year survival rate of 90% for stage 1 CRC and 10% for stage IV CRC was found in research studies conducted over a period of five years. Physical inactivity, a diet high in processed meats, smoking, being overweight, and abusing alcohol are some of the most common risk factors and primary causes of colorectal cancer. A person having a chronic inflammatory bowel illness, type 2 diabetes, or a genetic disorder like lynch syndrome has an increased risk of CRC and a family history of the disease (46). It is possible to treat CRC in stages 0 through 1, 2, and 3, but it is rare to treat stage IV(**Table 1**), which can be managed depending on the illness’s rate of growth and spread (47).

**Table 1:** Different stages of cancer along with its progression and the current treatment approaches available for Colorectal Cancer.

Stages of Cancer	Progression of each stage	Current Treatment Approaches for CRC	References
Stage 0	The earliest phase of CRC, also known as carcinoma *"in situ"* or intramucosal carcinoma, denotes that the disease has not yet spread through the colon or rectum wall and is present only in the mucosa (the moist tissue lining the colon).	Local treatments: Surgical resection	(39)
Stage I	Although it has penetrated the mucosal (second or third) layer of the colon, the cancer is still present in the inner lining. But it is still not been spreaded in the surrounding lymph nodes nor any distant sites have been affected by it.	Local treatments: Surgical removal of the cancerous polyp or partial colectomy of the tumour and local lymph nodes	(40)
Stage II	The colon or rectum's outer walls have been affected by cancer, which has spreaded and connected into other surrounding tissues or organs. But it hasn't migrated to local lymph nodes or distant regions.	Local and systematic treatments: Chemotherapeutic treatment (5-FU, leucovorin, oxaliplatin, or capecitabine); surgical excision without chemotherapy	(41)
Stage III	In stage 3, colon or rectum has developed cancer, or there are tiny tumour deposits in the fat surrounding the colon or rectum. It hasn't spread to distant sites.	Local, systematic, and combined treatments: Adjuvant chemotherapy, such as FOLFOX (5-FU, leucovorin, and oxaliplatin) or CapeOx (capecitabine and oxaliplatin), & is administered after surgery. For some advanced colon cancers, neoadjuvant chemotherapy, radiation therapy, and/or chemotherapy are options for patients who are not sufficiently stable for surgery.	(42)
Stage IV	Cancer has metastasised to distant sites and has been carried through the lymph and blood systems to distant parts of the body. The lungs and liver are the organs most likely to experience metastases from colorectal cancer.	Local, systematic, and combined treatments: Radiation therapy, chemotherapy with 5-FU, LV, and irinotecan ( FOLFOIRI), FOLFOX, CAPIRI (capecitabine and irinotecan), CAPOX, 5-FU with LV, irinotecan, capecitabine and Trifluridine plus Tipiracil (Lonsurf), immunotherapy with Pembrolizumab (Keytruda) or Nivolumab (Opdivo), targeted therapies	(43)

## Screening methods for colorectal cancer

CRC can be detected using a variety of screening assays, each with its own set of advantages and disadvantages. There are several factors that go into determining which screening test is best for a patient or clinician, including their perceptions and preferences. The actual positive rate is the percentage of patients who get a positive result from a screening test, which is the essential quality to look for in a screening test (48). In addition to sensitivity, but less so than specificity, which is the percentage of patients without disease who have a negative result, is significant, but less so than specificity (also known as the true-negative rate). The accuracy of a test is defined by its combination of sensitivity and specificity, which are typically traded off against one another based on the clinical scenario. Sensitivity is preferred over specificity when a serious or grave consequence of failing to detect a lesion or disease state is at stake. Specificity is preferred to sensitivity when the risk of overtreatment is the greatest (49). The use of a single test with both high sensitivity and high specificity is recommended in many screening applications. False-positive results would lead to excessive worry and follow-up, whereas false-negative results would leave CRC undiscovered. High precision is therefore crucial in the search for CRC. Repeatability and precision are other important considerations for the test. Obtaining high levels of cooperation from those who require screening is essential for a good screening program; as a result, the test must be acceptable to the individual. The test should be simple to administer and use, accessible, cost-effective, and safe in order to encourage a high participation rate in screening activities (50). CRC screening approaches, their advantages and disadvantages as well as where they are most successful in the CRC formation process, have been outlined here.

## Current treatments available for colorectal cancer

Surgery, chemotherapy, and radiation are among the most common types of treatment. Research into colorectal cancer has resulted in significant changes in treatment. In the past, Surgery, chemotherapy, and radiation were among the most common treatment modalities. There has been an increase in the number of therapeutic options for both local and advanced diseases as a result of a better understanding of pathophysiology (51). Patients can get a wide range of treatments, including endoscopic and surgical excisions, downstaging preoperative radiation, and systemic therapy, as well as major surgery for local and metastatic illness. Cancer patients who receive systemic chemotherapy and multimodal treatment are more likely to be cured or live longer than those who do not receive these treatments. Adjuvant and neoadjuvant CRC treatment are the two major treatment types. Neoadjuvant therapy, on the other hand, refers to treatments that are administered prior to the major cancer treatment, such as surgery (52). Neoadjuvant therapy has the ability to eradicate early metastases, hence reducing the severity of the cancer and reducing the likelihood of surgical complications. Surgical treatment is tailored to each individual patient and tumor and attempts to maximize survival and minimize recurrence risk. At the moment, there are a wide variety of surgical instruments and novel surgical techniques, such as minimally invasive surgery, being researched. Completely removing the tumor as well as its surrounding mesentery is a primary goal of CRC surgery (53). Patients who are unable to have this surgery due to the tumor’s location or invasion of the sphincter complex may benefit most from abdominoperineal surgery. Systemic treatment for cancer, chemotherapy involves the use of chemotherapy medications (54). Chemotherapy is usually administered orally or intravenously. 5-FU, oxaliplatin, irinotecan, and capecitabine are some of the chemotherapies used to treat CRC. When it comes to CRC treatment, the first line of defense is typically chemotherapy. 5-FU is the most commonly used chemotherapeutic medication in the treatment of CRC. It has been shown that 5-FU inhibits thymidylate synthase and has an anti-CRC function because it prevents the conversion of deoxyuridine to deoxythymidine. Chemotherapy’s adverse effects, which can range from nausea and vomiting to dry mouth and tongue to numb hands and feet to hair loss and reduced red blood cells, are well-known. Because of this, patient’s quality of life declines and can lead to the termination of chemotherapy treatment due to intolerance. Chemotherapy-induced side effects are currently not alleviated by any single drug. As a result of the advent of immunotherapy and targeted therapy, the cure rate and quality of life for patients with CRC have increased. Some tumours have responded well to immunotherapy treatment. Invasion of surrounding tissues by tumor cells in the tumor microenvironment (TME) is possible, as is metastasis via blood and lymphatic arteries (55). Therefore, it is essential to understand the TME’s immune status and investigate the distribution and activity of immune cells in order to increase the efficacy of immunotherapy in cancer. Multiple cell types in solid tumor tissues, such as malignant, innate, and adaptive immune system components including fibroblasts and endothelial cells and fibroblast-endothelial interfacial cells, contribute to the inflammatory and immunological condition of tumor tissues through cell-to-cell contact. It is possible to eradicate tumor cells from the body using the adaptive immune system and the natural immune system. As a result, tumor immunotherapy boosts the immune system’s ability to fight cancer by reducing immunosuppressive elements in the tumor microenvironment. Boosting the activity of T cells is the first step. Inflammatory checkpoint inhibitors can boost T cell responsiveness. immunosuppressive checks include CTLA-4, PD-L1, OX40, and Lag3. For example, nivolumab can inhibit CTLA-4 (56).

Targeted therapy has made a big difference in the success rate of CRC patients in recent years. Precision, efficiency, and low toxicity are the hallmarks of targeted therapy. The quality of life of CRC patients increases as a result of targeted therapy. Targeted drug research is the primary focus of the development of targeted therapy. Tumorigenesis, development, survival, or anti- tumor immunity are the primary goals of targeted treatments. For anti-cancer effects, targeted medications can interfere with these molecule’s functions and inhibit their signaling pathways (57). According to their method of action, CRC-targeted medicines can be categorised into three groups. For example, Cetuximab and Panitumumab inhibit tumor cell development by targeting tumor cell growth signaling pathways. Second, tumor growth-microenvironment-targeting medications like bevacizumab and regorafenib restrict tumor cell blood supply.

Traditional Chinese medicine, on the other hand, is becoming increasingly significant in modern medicine and cancer therapy. Traditional Chinese remedies have been demonstrated to have curative effects on colorectal cancer. Colorectal cancer has been discovered to respond well to a number of natural medicines (58). In Scutellaria baicalensis Georgi, Baicalein is one of the naturally occurring active components. Baicalein’s anti-inflammatory and anti-tumor properties are well-documented. A study found that baicalein was effective in the treatment of CRC in humans. Inhibiting colorectal cancer growth can be accomplished by baicalein’s ability to modulate gene expression. HT-29 and DLD1 cell growth, migration, and invasion are all inhibited by baicalein treatment. Hedyotis diffusa Willid (HDW) is an effective Chinese herbal remedy for treating colorectal cancer. This is according to JIUMAO LIN’s research, which states that HDW can suppress CRC by affecting the STAT3 pathway (59). HDW possesses antiangiogenic activity, which is essential for cancer growth and progression. The ability of EEHDW to prevent cancer growth has been established both *in vivo* and *in vitro*. There are multiple CRC-related signaling pathways that EEHDW inhibits and controls the expression of, among other things. The primary active ingredient in ginseng is ginsenoside, which has been shown to be effective against colorectal cancer. Many kinds of cancer, including CRC, are influenced by the epithelial-mesenchymal transition (EMT). Antiangiogenic therapy has been used successfully to treat colorectal cancer because tumor tissue contains a large number of blood vessels (60). This ginsenoside possesses anti-vascularization properties, can prevent tumor growth and metastasis and can make cancer cells more sensitive to treatment.

## Nano construct preparative methods

The efficacy of many medications and therapeutically active molecules like nucleic acids and proteins can be enhanced by using nanocarriers, while the risk of harmful and side effects is reduced (61). It is possible to shield therapeutic compounds from degradation, manage their release, bypass biological barriers, and target specific locations of action with biodegradable nanoparticles (NPs) (62). By altering interactions with the biological environment, the physicochemical features of nanoparticles can influence the biodistribution and pharmacokinetics of medications. For intravenous delivery, the size of the nanoparticles is particularly important because opsonin’s (plasma proteins) adsorb onto the particles, resulting in the macrophages of the RES being able to recognize and remove them from the bloodstream (63). NPs with a diameter less than 80 nm were demonstrated to be more difficult to remove from the bloodstream than larger particles, which had a higher concentration of plasma proteins on them. The spleen’s ability to filter out NPs and the hepatic parenchyma’s ability to trap them both depended on their size. Cancer therapy can benefit from the so-called EPR, the leaky vasculature of some solid tumours, combined with weak lymphatic drainage, may result in a selective accumulation of colloidal carriers within the target tissue, allowing for more effective treatment (64). Many human tumours endothelium has an effective pore size ranging from 200 nm to 600 nm, according to research. Particles must be fewer than 200 nm in diameter to benefit from the EPR effect, and much more preferable less than 100 nm. A diameter of more than 10 nm, on the other hand, usually prevents the diffusion of NPs through artery endothelium, which minimizes negative effects in healthy tissues (65). The mechanisms of NP internalization, phagocytosis, or endocytosis, are impacted by size at the cellular level as well (66). As a result, precise control of NP size and size distribution is required to provide effective and secure drug delivery. The detailed discussion of various preparative methods as described below:

### Nanoprecipitation method

**Figure 2:**
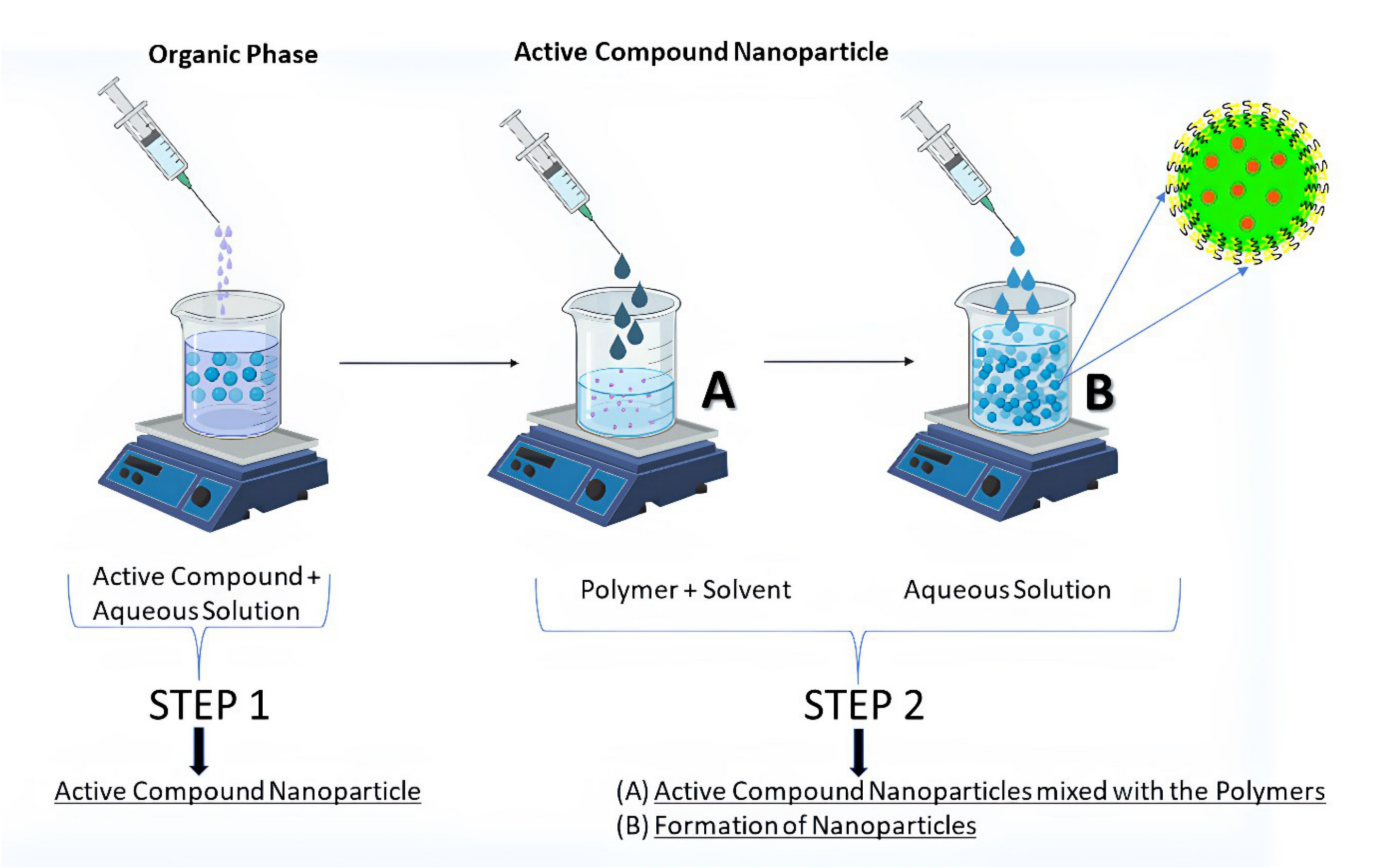
Formation of nanoparticles by the two-step nanoprecipitation method.

Fessi et al. awarded a patent in 1989 for nanoprecipitation. For hydrophobic drug compounds, it was mostly used after its invention (nanocapsule or nanosphere forms) (67). Numerous biodegradable polyesters such as PLA (Polylactide acid), PLGA (Poly (d,l-lactic co-glycolic acid)), and PCL (Poly-caprolactone) have been employed to achieve this goal, among them PLA, PLGA, and PCL. According to Fessi et al., the preparation of solvent and nonsolvent phases is necessary before the addition of one phase to the other under moderate magnetic stirring in this process. NPs can be suspended in water by evaporating organic solvents at room temperature or using a rotavapor (68). Ethanol, acetone, hexane, methylene chloride, and dioxane are the most common nanoprecipitation solvents. Water predominates in the non-solvent phase. Nonsolvent phases may also be supplemented with hydrophilic excipients. Particle size and surface morphology can be determined using TEM, SEM, or dynamic light scattering (DLS). The physical features of nanoprecipitates, such as their size, drug encapsulation effectiveness, and so on, are influenced by a wide range of parameters in nanoprecipitation. Using nanoprecipitation (**Figure 2**), the most prevalent breakthroughs in the pursuit of polymer, lipid, and hybrid particles involve submicron and nanometric scales of nanoprecipitation. This technique is simple, energy-efficient, and adaptable. Nanocarrier’s *In vivo* behavior is becoming better understood since industrial-scale production necessitates better control and standardization of operations. As a result, the technique and the starting materials used to make them have been improved to meet these needs. Particles with hydrophobic and hydrophilic molecular entrapment or behavior as stealth carriers can be produced using sophisticated devices with sizes less than 100 nm, and the procedure has been fine-tuned through chemical modification of polymers or careful definition of working conditions (69). Even more interesting is the invention of hybrid nanoparticles, which are able to offer substantial drug loadings, long-term drug-release patterns, and improved pharmacokinetic features. For the production of safe particles, nanoprecipitation appears to be a viable option regardless of the carrier material being used. Even when solvents with a high level of inherent toxicity are used, the positive results of safety testing show that they can be used in the pharmaceutical industry.

Pereira et al. described that PLGA-PEG NPs were generated by nanoprecipitation and loaded with paclitaxel (PCX), after which they were surface-functionalized with a monoclonal antibody targeting the carcinoembryonic antigen (CEA) of intestinal epithelial cells. Two intestinal cancer cell lines, Caco-2 clone and non-Caco-2 clone SW480, were used to evaluate the nanoparticles’ physical and chemical characteristics, cytotoxicity, and ability to target. Nanoparticles with a diameter of 200 nm and close to charging neutrality were successfully produced, encasing up to 99 percent of paclitaxel. Further development of functionalized nanoparticles showed that they were non-cytotoxic to intestinal cells. Flow cytometry confirmed the capacity of functionalized nanoparticles to target Caco-2 CEA-expressing cells, unlike SW480 cells. PCX nanocarriers with CEA-targeting antibody were successfully generated as nanoparticles and interacted with CEA-expressing cells. These particles can be exploited as targeted systems for CRC therapies because of their unique interaction (70). Ahmad et al. reported the development of amino cellulose-grafted polymeric nanoparticles containing LCS-1 for synthetic lethal targeting of checkpoint kinase 2 (CHEK2)-deficient HCT116 colon cancer cells to surpass the limitations associated with the solubility of LCS-1 (a superoxide dismutase inhibitor). To create nanoparticles containing LCS-1, amino cellulose (AC), a biocompatible and biodegradable hydrophilic polymer, was grafted onto polycaprolactone (PCL). This study utilized LCS-1-loaded PCL-AC NPs to suppress CHEK2-deficient HCT116 CRC cells using the synthetic lethal interaction between SOD1 and CHEK2. PCL-AC nanoparticles were also examined in terms of their size, cellular absorption, and cell survival after the development of protein coronas. An LCS-1-loaded NPs were analyzed for their polydispersity index (PDI), zeta potential (ZP), and morphological properties by transmission electron microscopy (TEM), scanning electron microscopy, and atomic-force microscopy. Confocal imaging showed that nanoparticles were taken up by HCT116 cells, as demonstrated by the cellular internalization. It was also shown that NPs were cytocompatibility because they did not harm hTERT and HEK-293 cells. When compared to colon cancer cells expressing CHEK2, the LCS-1-loaded PCL-AC NPs were up to 240 times more selective in their ability to kill CHEK2-deficient cells. As a result, the protein corona-coated nanoparticles of PCL-AC NPs were shown to be incubated in human and fetal bovine serum by SDS-PAGE analysis. Hydrodynamic diameter increased slightly for PCL-AC nanoparticles coated with corona, and this was validated by TEM. There was also cell uptake and no harmful effects on hTERT cells when PCL-AC NPs were coated with a coronal layer. By developing a nano formulation of LCS-1, researchers hoped to increase its ability to kill colorectal cancer cells with CHEK2 lack (71).

### Ionotropic gelation method

**Figure 3:**
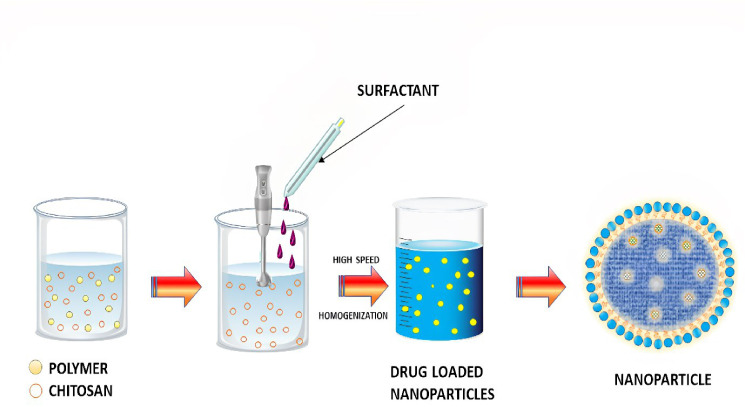
Schematic representation of Ionic gelation method.

It is one of the simplest and most cost-effective methods for ionotropic gelation in the laboratory. Nanoparticles and microparticles made of polymeric materials are being used in the hunt for novel and better treatments (**Figure 3**). There are numerous advantages to these formulations due to the inclusion of biocompatible and biodegradable polymers. As a result of the technique’s simplicity and mildness, the complexation of chitosan (CS) nanoparticles with two oppositely charged macromolecules has garnered considerable interest. As a result, electrostatic cross-linking, rather than chemical cross-linking, has been used to minimise probable toxicity and other negative effects on the reagents. CS can interact electrostatically with polyanion tripolyphosphate (TPP). It was after the report of Bodmeier et al. that many researchers began to investigate the possible pharmacological use of the TPP–CS complex. It is possible to obtain the cation of CS by dissolving CS in an aqueous acidic solution in the ionic gelation technique. To make a polyanionic TPP solution, add this solution dropwise while stirring continuously. It is possible to cross-link chitosan nanoparticles by reacting with the negatively charged phosphoric ions of TPP because the chitosan molecules have an abundance of the NH3 group. Cross-linking and hardening processes may aid to maintain drug release by evaporating water from the particles throughout this period. The solution, aggregation, and opalescent suspension all occurred in making the nanoparticles (72). There is an end in sight at this point. Insulin-loaded CS nanoparticles were made by first combining insulin with TPP solution and then adding this to CS solution while stirring constantly. Both CS hydrochloride salts, with molecular weights and deacetylation levels varied, were used to create nanoparticles. CS and TPP concentrations were changed so that the CS/TPP ratio was equal to 3.6:1. A positive surface charge of between +34 and +45 mV was found on the chitosan nanoparticles produced. As a result of this strategy, insulin loading was adjusted up to 55%. Due to the gelation of protonated amino groups of CS1, the method’s effectiveness was contingent on the deacetylation of CS. For example, peptide and protein formulations are shown to improve oral bioavailability in a number of ongoing studies. With the addition of nanoparticles of bioadhesive polysaccharide CS, it appears that their intestinal absorption is enhanced (73). P53 polyplex-loaded enteric-coated calcium pectinate microbeads for oral gene delivery were developed and tested by Bhatt et al. as a potent new treatment option for CRC. In CRC, mutations in the p53 gene are a primary event and an important target for gene therapy treatment. Colon cancer cell lines were transfected with polymethacrylates-based non-viral vectors to test its ability to complex, protect, and transfect p53. At varied N/P ratios, polyplexes were formed by the complexation of cationic polymer with anionic pDNA. Ionotropic gelation was used to create p53 polyplex-loaded calcium pectinate (CP) microbeads covered with Eudragit^®^ S100. Enteric-coated CP microbeads were shown to protect the release of p53 polyplex in the upper GIT in *in vitro* release tests with less than 10% release. Polymethacrylate carriers have been shown to effectively transfect pDNA in both *in vitro* and *in vivo* investigations in rat cell lines. Results from an *in vivo* gene expression investigation demonstrated the potential of enteric-coated calcium pectinate microbeads to transfer pDNA to the colon of rats. As a result, calcium pectinate microbeads covered with enteric-coated calcium pectinate released p53 polyplex in the colon and may be an effective alternative to CRC therapy (74). Motawi et al. created Cromolyn chitosan nanoparticles (CCSNPs) using an ionic gelation process to enhance bioavailability and tested for their anticancer properties in a dimethylhydrazine-induced model of colorectal cancer in rats. To promote colon cancer in the rats, groups were separated into seven and given dimethylhydrazine for 16 weeks; group 1 was given a normal control, group 2 cromolyn control, and group 3 CCSNPs control. Protective therapies for groups 5–7 included cromolyn solution, non-medicated NPs, and CCSNPs. Dimethylhydrazine was found to be ineffective in reducing tumor-signaling molecules and the number of abnormal crypt foci in comparison to optimal CCSNPs (size 112.4 nm, charge 39.9 mV, encapsulated 93.6% cromolyn, displayed a sustained drug release pattern over 48 h) Cromolyn solution, on the other hand, was found to have a protective impact on colon cancer cells that was enhanced by CCSNPs’ ability to improve tumor pathology. Finally, in colorectal cancer tissue, CCSNPs improved tumor pathology and malignant oncogenic signaling molecules. CCSNPs, on the other hand, may offer a novel method of protecting against colorectal cancer treatment. Additionally, the anticancer properties of cromolyn were improved when it was encapsulated in chitosan nanoparticles (75).

### Solvent evaporation method

**Figure 4:**
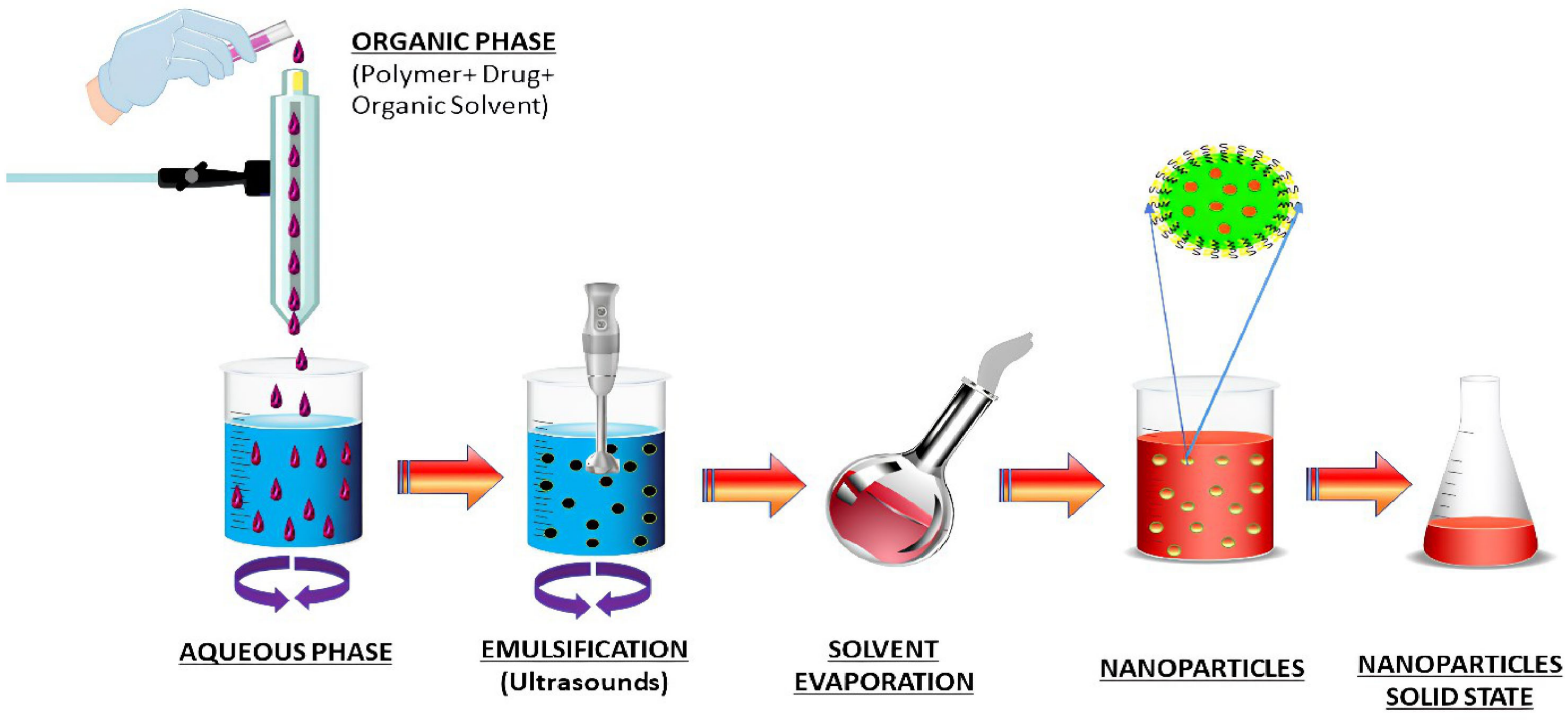
Schematic diagram of nanoparticles preparation by emulsification solvent evaporation method.

The emulsion solvent evaporation technique is a method to develop NPs and nanocapsules that is well suited for uses that need high purity and low toxicity materials, including biomedicine or electronics. An organic solvent such as chloroform, acetone or ethyl acetate is used to dissolve premade polymers such as PLA or PLGA. An emulsion of an oil and water mixture is formed by dissolving the medication in a polymer solution, which is then transferred to an aqueous phase containing a surfactant such as polyvinyl alcohol (PVAL). It is possible to speed up the evaporation of organic solvents by extending the homogenization process (76). An ultracentrifuge collects the nanoparticles at the end of the homogenization stage. The **Figure 4** depicts a schematic representation of this approach. Polymer-to-organic solvent ratio, type of organic solvent, and homogenization time and speed can all be manipulated to produce the desired particle size and other characteristics. The emulsion–solvent evaporation process has been mysteriously understudied for a long time. Basically, a polymer dissolves in an excellent solvent, which is emulsified into an aqueous solution that contains a surfactant. Nucleation of the polymer occurs on the water–solvent interface due to the sluggish evaporation of the polymer-solvent. The rate of evaporation is determined by the solubility of solvent in the continuous phase; hence temperature and the type of the solvent are critical factors. Gas chromatography or NMR spectroscopy can be used to monitor the evaporation process, which is normally completed within a few hours. Particle hardening is affected by evaporation, given that the solvent in the dispersed phase is largely evaporating from a saturated continuous phase and its diffusion rate into the dispersed phase is much faster than the evaporation kinetics of the solvent. Wang and Schwendeman found that the rate-limiting phase for solvent mass transport is dependent on the solvent’s characteristics in an experiment, including dichloromethane, ethyl acetate, and acetonitrile as solvents. While ethyl acetate and acetonitrile were discovered to be gas-side limited, chloromethane was found to be liquid-side limited. Temperature and impeller diameter had the greatest impact on the pace at which water evaporated. Particle hardness profiles might be anticipated and determined without having to monitor the conc. of polymer in the solvent at any given moment, but rather by measuring the concentration of solvent and knowing its permeability coefficient at the liquid–air interface. The dispersions can be dialyzed to remove low molecular weight polymer and freeze-dried after the solvent has been evaporated (77). Docetaxel (Doc) and LL37 peptide polymeric nanoparticles (Doc+LL37 NPs) were coencapsulated in a thermosensitive hydrogel system by Fan et al. to create a biodegradable and injectable drug delivery system for the treatment of colorectal peritoneal cancer. Biodegradable Doc+LL37 NPs were first prepared via a solvent-evaporation approach including a water-in-oil-in-water double emulsion of PLA-Pluronic L35-PLA. This was followed by the preparation of a biodegradable and injectable thermosensitive PLA-L64-PLA hydrogel with reduced sol–gel transition temperatures near body temperatures. The Doc+LL37 NPs produced by the PLA-L35-PLA copolymer were found to be spherical using TEM. That Doc and LL37 were correctly packaged was verified using Fourier-transform infrared (FTIR). Doc was found to be encased in an amorphous X-ray diffraction pattern. HCT116 peritoneal carcinomatosis *in vivo* was greatly slowed down, and animals bearing the tumor had a longer survival time after receiving an intraperitoneal injection of Doc+LL37 NPs–hydrogel (78). The colon-specific DDS developed by Dang et al. was developed as matrix-type microspheres by solvent evaporation utilizing the ethyl cellulose (EC), cellulose acetate phthalate (CAP), and eudragit L 100-55. The drug concentration, particle size, bulk density, and angle of repose of microspheres were all measured. Drug conc. varied between 74.49% and 91.50% depending on the polymer and polymer ratio of the microcapsules, which ranged from 228 to 608 micrometer’s. There was a good flow property of 1.2 g/ml mean bulk density, and a free-flow property of 40 angle of repose. Except for the microspheres containing CAP, and EC which had a rough and porous surface, all of the microspheres were spherical and nonporous. Eudragit L 100-55 microspheres combined with other polymers provided superior sustained release (78.9 and 76.6 percent after 8 hours for formulations F4 and F5, respectively) than the other microsphere formulations tested. A 1:2:1 ratio of diclofenac sodium, EC and CAP in microspheres shows the maximum drug content, good flow characteristics and surface shape, and promising drug release for colorectal cancer treatment using diclofenac sodium microspheres (79).

### Salting out

Bindschaelder et al. patented the salting-out process in 1988, and it describes how to make a pseudo-latex (a colloidal dispersion) out of a water-insoluble polymer. A premade polymer dispersed in an aqueous media yields pseudo-latexes, which are colloidal systems with particles with an average size of less than one millimeter. Pseudo-latexes can be made from a variety of polymers using the salting-out approach. In addition, low-toxicity solvents like acetone or ethanol can be used to synthesize drug-loaded PNPs, in contrast to other approaches. The first step in the salting-out process is the mechanical mixing of two phases to create an oil-in-water emulsion. The water-miscible solvent is used to dissolve a water-insoluble polymer and an active ingredient in the oil phase, while colloidal stabilizers and salting-out agents are used in the aqueous phase. Sodium bicarbonate, the salting-out agent in the water, blocks solvent diffusion. It’s then diluted with enough clean water to drop the salting-out agent’s concentration below a threshold, allowing the organic solvent to rapidly permeate into the water and causing interfacial turbulences and PNPs. Distillation at lower pressure removes the solvent from the PNP suspension. The salting-out agent is removed by ultracentrifugation and repeated washing processes and the leftover stabilizer. Cross-flow filtering can be used to remove the salting-out agent and the solvent (80). PNPs can be formed by salting-out in a manner similar to that postulated for the solvent displacement approach, despite the lack of research into the mechanism. An emulsion spreads violently when it is mixed with water because of the quick mutual miscibility of the solvents. Nanometric-sized solvent droplets are snatched off the interface. The surfactant ingredient quickly stabilizes these droplets, causing the polymer to aggregate into nanoparticles after complete solvent diffusion. The salting-out technique has a number of advantages over the solvent displacement method, including the ability to produce high-concentration and stable dispersions because of the inclusion of substantial amounts of polymer. When lipophilic medicines are utilized, high doses of medication can be integrated with good entrapment efficacy. Another advantage is that it may be easily scaled up to produce larger nanoparticles with the suitable selection of agitation settings (81). Allemann et al. developed aqueous polymeric nano dispersions by a reversible salting-out process. Surfactants and chlorinated solvents were avoided in the emulsion approach used to create the polymeric nanoparticles as aqueous dispersions. PVAL is a viscosity-enhancing agent and stabilizer that is added to an acetone solution of the polymer under continuous stirring in order to form the final product. A salting-out technique prevents acetone from combining with water in the saturated aqueous solution. Nanospheres are formed when water is added to an oil-in-water emulsion in a sufficient amount to allow for the complete diffusion of the acetone into the water phase. PVAL conc. And its type in the aqueous phase were also altered as well as stirring rate and internal/external phase ratio during the manufacturing process (82). Sengel et al. prepared nanoparticles by using salting-out and emulsion-evaporation steps. It was shown that PLGA and PVA molecular weight differences had an impact on the NPs’ physicochemical qualities. Over the course of three months, meloxicam’s stability in NPs was evaluated. Assays for cell uptake and viability were performed using the HT-29 cell line, which expresses COX-2. Size range was from 170–231 nm; the PDI was lower for NPs with a spherical form and a negative ZP. The physical stability of NPs produced with high molecular weight PLGA was demonstrated for three months at 4°C. When the polymer and the emulsifier increased in molecular weight, it also decreased meloxicam’s *in vitro* release rate. It was found that meloxicam-loaded NPs were cytotoxic to HT-29 cells at 800 M. Coumarin-6-loaded NPs were highly absorbed by cancer cells. To treat colon cancer, the PLGA NPs created in this study could be an effective DDS for meloxicam (83).

## Cellular mechanism of nanoparticles

**Figure 5:**
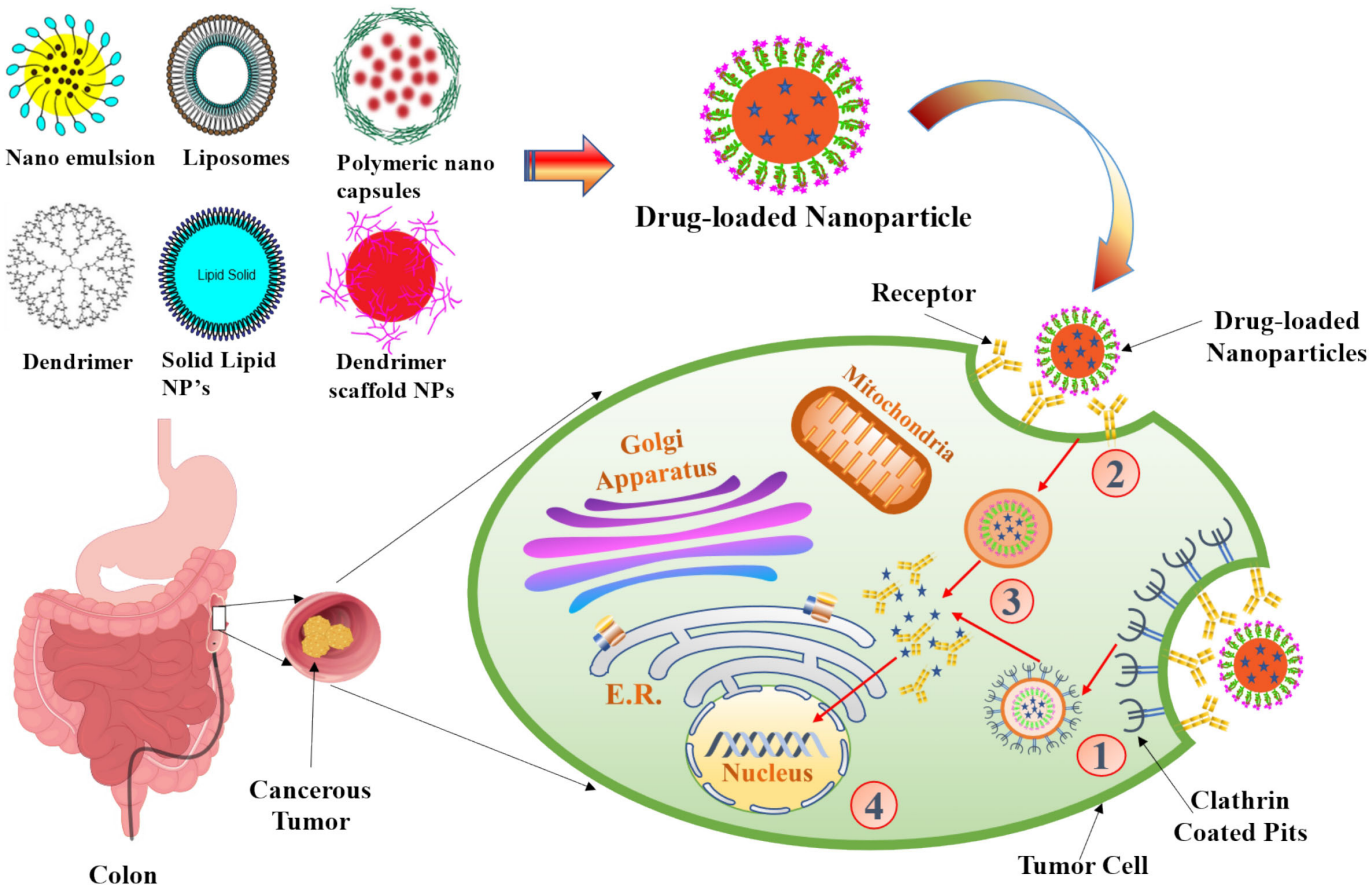
The scheme of endocytosis of the nanoparticle modified by antibodies that recognize cancer markers at the cell membrane. (1) Receptor (aptamer or antibody) on the drug-loaded nanoparticles recognize the clathrin-coated pits in the tumor cell and bind to it. (2) Phagocytosis of drug-loaded nanoparticles facilitates the transport of the carrier into the tumor cells. (3) An endocytic vesicle is formed. (4) Endosome induces the release of drug from the nanoparticles and penetrates into the nucleus. Representation of the different kinds of NPs included in this review is also shown.

Particles enter cells via the endocytosis route, that includes phagocytosis and pinocytosis, in biological systems (84). Nanoparticles with diameters of less than 200 nm are engulfed by micropinocytosis (**Figure 5**), which can take place in one of the following ways: with or without clathrin/caveolae (85). Phagocytosis/micropinocytosis takes in large particles. Both the pathways are distinct in their mechanisms and strictly regulated at the molecular level. The route that NPs take inside cells determines the intracellular nanoparticle transport its biological and therapeutics outcome (86). The interaction of NPs with the target cells can be essential or destructive to the organism as a whole, depending on the intended outcome as required for the particular application. NPs interact with serum and extracellular matrix (ECM) proteins as they enter the human, creating a “protein corona” surrounding them (87). Some of the NPs are recirculated back into the extracellular space via the clathrin-mediated mechanism (88). The cellular uptake of NPs (20–100 nm) is mediated by caveolin pathway, whereas the cellular uptake of submicron particles (100–350 nm) is mostly mediated by the clathrin-mediated pathway (89) Based on studies indicated, the co-localization of caveolin-1 proteins over internalised NPs discovered in the caveolae and caveosomes suggested that nanoparticle uptake could occur by caveolae-mediated endocytosis (90, 91). When chemotherapeutic drugs are delivered in nanoparticle form, their fate in the bloodstream is determined by their physicochemical qualities, as well as the elemental compositions of those nanoparticles (92). Kou et al. have revealed a number of different mechanisms by which nanoparticles can be absorbed into cells. Nanoparticles should be made from non-toxic or biocompatible materials to avoid hazardous effects, in addition to their physicochemical features (93). Several caveolin and clathrin-independent endocytosis pathways, such as Arf-6, Rho-A (or IL2Rb-dependent pathway), flotillin, or CDC42 (CLIC/GEEC)-dependent endocytosis, exist in addition to the mechanisms mentioned above; however, the present review will not go into further detail on these pathways because they do not significantly contribute to cellular NP uptake.

## Role of nanomedicine in targeted delivery for treatment of CRC

In the case of CRC, targeted therapy for nanoparticles has significantly improved the overall survival in patients with the condition. The development of checkpoint inhibitors is accelerating at an unparalleled rate due to the increasing efficacy of therapies like the anti-EGFR (epidermal growth factor receptor) agent (cetuximab) and the anti-angiogenesis agent (bevacizumab), which target multiple immunological pathways (58). Targeted delivery, which can be attained by passive or active targeting, which tries to precisely target specific cancer cells. Passive targeting uses the enhanced permeability and retention (EPR) effect, whereas active targeting involves conjugating small molecules, aptamers, peptides, and antibodies. Targeted drug delivery, as compared to free drug, aids in lowering the toxicity in normal cells, protecting drugs from deterioration, prolonging half-life, increasing loading capacity, and enhancing solubility (94, 95). We included examples of published *in vitro* and/or *in vivo* nanoparticle targeting ligands and their receptors for nanomedicine applications in **Table 2**.

**Table 2:** Examples of different types of nanoparticle targeting ligands and their receptors along with its active agents.

Nanoparticulate Drug Delivery System	Drug (Active Agent)	Ligand	Receptor	Size Range (nm)	Sources
PEGylated hollow mesoporous ruthenium nanoparticles	[Ru(bpy)2(tip)]2+, RBT	SS-Fc	Carcinoembryonic antigen	110	(93)
Hyaluronic Acid–Doxorubicin nanoparticles	Doxorubicin	Hyaluronic acid	RHAMM, CD44	175	(58)
Cyclodextrin-based host–guest complexes	Regorafenib	Mannose	Mannose receptor	100	(94)
Cationic liposomes	ESC8, anti-Hsp90 plasmid	Dexamethasone	Glucocorticoid receptor	251	(95)
Polymersomes	Doxorubicin	Transferrin	Transferrin receptor	72	(96)
Self-assembled nanoparticle	Bortezomib	Folate	Folate Receptor	196	(97)
PLGA nanoparticles	5-Fluorouracil	Wheat germ agglutinin	N-acetyl-D-glucosamine, sialic acid	156	(98)
Nanoscale metal organic framework	Talazoparib, temozolomide	Fucoidan	P-selectin	84	(99)
PAMAM dendrimers	Celastrol	EpCAM aptamer	Epithelial cell adhesion molecule	300	(91)
Human serum albumin nanoparticles	5-Fluorouracil	LRP-1 targeting peptide	Lipoprotein receptor-related protein-1	208	(100)
Lipidic core nanocapsules	Thymoquinone	Anisamide	Sigma receptors	217	(101)
Mesenchymal-stem-cell -derived exosomes	Doxorubicin	MUC1 aptamer	MUC1	50	(102)
Silica nanoparticles coated with chitosan	AntimiR-21, Doxoubicin	AS1411 aptamer	Nucleolin	87	(103)
Nanoparticles	Paclitaxel, chlorin e6	Tumor-homing peptide tLyp-1	NRP-1	107	(104)
PLGA and PEG-based polymeric nanoparticles	5-Fluorouracil	Anti-EGFR mAb	Epidermal Growth Factor Receptor	252	(92)

### Passive targeting nanoparticles

The term “passive targeting” refers to nanoparticles that lack specific surface targeting ligands (96–98). Because the malignant tumor lacks functional lymphatic vessels and has a wide gap between tumor endothelial cells. The effect that makes NPs accumulate in the malignant cells is called as EPR (99, 100). In the research study, resveratrol and oxaliplatin were encapsulated into N, O-carboxymethyl chitosan NPs using emulsification crosslinking and ionic crosslinking, respectively. In comparison to the free drug, the NPs improved the solubility, stability, and EPR effect of resveratrol and oxaliplatin, leading to higher anti-CRC action in SC tumor-bearing rats (101).

The non-specific nature of the interactions between NPs and cells is indicated by passive targeting and the absorption of nanoparticles in both healthy and diseases state may be facilitated by these non-specific interactions (102). In recent years, researchers have gradually come to understand how the tumor-targeting mediated by EPR is extremely heterogeneous. In order to improve the targeting capability of NPs based on EPR, it should be combined with different targeting mechanisms (103).

### Active targeting nanoparticles

In active targeting, the surface of nanoparticles is modified with the targeting ligands to enable specific interaction along with the binding of these nanocarriers to cell (103). Cancer cells produce macromolecules and overexpress certain receptor types, which promote the proliferation cancer cells and its surrounding tissues through autocrine or paracrine pathways (104). In recent times, the active targeting of nanoparticulate targeted DDS for CRC has primarily utilized the receptor-ligand binding strategy, which involved many highly expressed receptors in CRC, including the EGFR, mannose receptor, CD44, epithelial cell adhesion molecule (EpCAM), nucleolin, v3 integrin receptor, folate receptor, carcinoembryonic antigen, hyaluronic acid receptor, glucocorticoid receptor, lipoprotein receptor-related protein etc (103). Ge et al. developed biological conjugates loaded with celastrol that could be captured by CRC overexpressed with EpCAM in order to decrease drug toxicity and enhance therapeutic efficacy. The conjugates were composed of dendrimers, PEG, and the EpCAM aptamer. The findings demonstrated that when exposed to biological conjugates, SW620 will experience significant apoptosis. Moreover, the biological combination demonstrated reduced toxicity in xenograft mouse and zebrafish models (105). Another example was to deliver anti-EGFR receptor-5-fluorouracil (5-FU) in which Sankha Bhattacharya developed PLGA-PEG-coated NPs, which could enhance the pharmacodynamics and distribution of the drug *in vivo*. Through the RES, the polymeric NPs composed of PLGA and PEG can inhibit opsonic action. These nanoparticles have significant therapeutic significance due to their quick and easy production processes of solvent emulsification and evaporation (106).

Integrating cell surface receptor-specific targeting ligands to nanoparticle surfaces may improve cellular interactions. Besides specifically targeting cancer cells, active targeting approaches can also exploit the distinctive characteristics of the tumor microenvironment. One approach involves exploiting the hypoxic microenvironment within tumours for targeted therapy. Hypoxia-activated prodrugs have been developed to release cytotoxic agents specifically in response to the low oxygen levels in tumor tissues (107). Cetuximab, when combined with chemotherapy, has demonstrated improved outcomes in patients with metastatic colorectal cancer. It’s worth noting that other targeted therapies, such as anti-VEGF agents (bevacizumab) and immune checkpoint inhibitors (pembrolizumab), are also being studied for colorectal cancer and utilize active targeting strategies to engage specific molecular targets in the tumor microenvironment (107).

However, precise targeting of cell populations *in vitro* and *in vivo* necessitate that the surface modifications of NPs can maintain its integrated design function. As a result of these findings, scientists now have the chance to develop nanoparticle surface patterns that can address the challenge to improve the interactions and its specific delivery between targeted cells and nanoparticles (86).

## Role of nanotherapeutic in the management of CRC

One of the three most frequent malignancies in the world, colorectal cancer is typically diagnosed in the middle or late stages, and the affected population is getting younger. Intensive chemotherapy is required, especially in individuals with late-stage CRC, although it might have undesirable side effects and unpleasant reactions because of drug resistance and damage to normal cells. Various types of nanoparticles have been explored for their potential in combating drug resistance in colorectal cancer. For example, polymeric NPs like PLGA NPs have been utilized to encapsulate conventional chemotherapy drugs such as 5-FU and oxaliplatin. These nanoparticles enhance drug stability, prolong drug release, and increase drug accumulation within cancer cells, thereby overcoming mechanisms of drug resistance (108, 109). Additionally, nanoparticles have the ability to deliver innovative therapeutic agents to address drug resistance. One approach involves loading small interfering RNA (siRNA) into nanoparticles to silence specific genes associated with drug resistance pathways. By silencing these genes, nanoparticles can sensitize cancer cells to chemotherapy and enhance their therapeutic response (110). Additionally, nanoparticles could be designed for combination therapy targeting multiple pathways involved in the immune system. For example, nanoparticles can simultaneously deliver therapeutic drugs and drug efflux pump inhibitors such as P-glycoprotein (P-gp). By inhibiting drug efflux mechanisms, nanoparticles improve intracellular antibody accumulation and overcome drug resistance (111). In conclusion, nanoparticles show the potential to resolve resistance to colon cancer by improving drug, improving drug stability and targeting cancer. Improved bioavailability and reduced adverse effects can be greatly attributed to research on targeted drug delivery, which enables the treatment of cancer without harming healthy cells (112). Small molecule medications can be carried and accumulated in tumor tissue using nanocarriers, a type of nanosystem. Nanocarriers are superior to free medications because of their excellent efficacy and low incidence of side effects. Protect the medication against degradation, limit renal clearance, boost half-life and bioavailability, and slow drug release rate with nanocarriers in addition (113). In this section, we emphasized that chemotherapeutics, targeted medicines, and natural medications are all influencing the creation of nano preparation for the treatment of CRC (**Table 3**).

**Table 3:** Detailed description of nano-vesicular based delivery system for the management of colorectal cancer.

Author (s)	Delivery system (s)	Preparative method	Drug (s)	Therapeutic Intervention	References
Bansal et al; 2016	Liposomes	Cast film method	Oxaliplatin	Folic Acid conjugated liposomes encapsulating Oxaliplatin were entrapped in alginate beads and subsequently coated with Eudragit-S-100 for efficient delivery to colon cancers. The study indicated that Eudragit coated alginate beads got into the colon of Balb/c mice between 4.20 and 4.50 h after oral administration. *In vivo* findings demonstrated that folic acid associated liposomes encapsulated in alginate beads supplied 2.82 ± 0.58 and 21.52 ± 2.76 μg L-OHP/g tissues in the colon and tumour after 12 h, showing its targeting capacity to colon and tumour.	(107)
Yang et al; 2011	Liposomes	Reverse-phase evaporation method	Oxaliplatin	An investigation was conducted on the efficacy of PEG-Oxaliplatin long circulating liposomes, to increase the therapeutic index of colorectal cancer. PEG-liposomes were able to form a stable bond with cells after only 2 hours, and the mean fluorescence intensity increased when the induction period was lengthened. Comparatively, 23.21 percent ± 3.38 percent, respectively, of the cells were apoptotic in the presence of PEG-oxaliplatin liposomes (P-ox-L) and free oxaliplatin liposomes (ox-L). Using *In-Vivo* Imaging, fluorescence imaging showed that PEG-liposomes specifically targeted tumour tissue. Compared to free (ox-L), (P-ox-L) PEG-liposomal L-oHP reduced tumour volume by 26.08 percent ± 12.43 percent and increased life span by 45.36 percent, respectively, after intravenous injections.	(108)
Soo et al; 2015	Liposomes	Extrusion technique		Polymeric nanoparticles and liposomes have demonstrated limited success in encapsulating resveratrol. Liposomes were used in this study to co-encapsulate both the lipophilic and hydrophilic components of the medicine by adopting a novel dual carrier strategy to build and optimise a novel drug carrier. The final formulation had a particle size of 131 nm, a polydispersity index of 0.089 nm, and a zeta potential of -2.64 mV. Nanoformulations released 100 percent of the medication in 24 hours, compared to a drug release profile of 40-60% for free resveratrol and standard liposomal formulations. It remained steady at 4°C for 14 days. Resveratrol was found to have an increased cytotoxicity profile when it was incorporated into liposomes rather than free resveratrol. The findings of the study shows that liposomal formulations containing pristine resveratrol and its cyclodextrin complex are a viable approach for improving hydrophobic chemotherapeutic drug distribution.	(109)
Chaurasia et al; 2015	Polymeric nanoparticles	Emulsification-diffusion-evaporation method	Curcumin	Curcumin bioavailability and anti-cancer activity *in vitro* and *in vivo* have been improved by the development of a novel polymeric nanoparticle. In terms of particle size and entrapment efficiency, curcumin encapsulated nanoparticles (CENPs) were found to be satisfactory. Compared to pure CUR, *in vitro* cytotoxicity experiments using 50% cell growth inhibition values showed a 19-fold reduction when CENPs were used. Oral administration of CUR as CENPs increased its oral bioavailability by 91- and 95-folds, respectively, compared to oral administration of pure CUR.	(110)
Tummala et al; 2015	Polymeric nanoparticles	Solvent evaporation emulsification method	5-Fluorouracil	The primary goal was to synthesise chitosan polymeric nanoparticles. Simulated GIT tract fluids were used for *in vitro* drug release investigations, with pH values ranging from 1.25 to 7.0. 5-FU nanoparticles with a drug:polymer ratio of 1:2 and 1:3 have shown improved particle size (149 nm and 138 nm respectively) and entrapment efficiency (48.12 ± 0.08 percent and 69.18 ± 1.89 respectively). Drug release from 5-FU after four hours is superior to that from non-enteric coated tablets, which released more than half of their medication before it reached the colon. Nanoparticles generated by this process employing a polymer with an optimum ratio can be used as a potential drug delivery mechanism for the effective delivery of the active pharmaceutical ingredient to colorectal cancers.	(111)
Yakati et al; 2022	Polymeric nanoparticles	Emulsion-solvent evaporation method	Paclitaxel	Polymeric nanoparticle delivery systems that target both cancer cells and endothelial cells should be developed, using CPKSNNGVC (CPK in short) as a nanoparticle surface-bound targeting ligand for cancer cells. Nanoparticles with a diameter of 215 ± 4 nm, a zeta potential of 12 ± 3 mV, and a concentration of 16–17.5 percent w/w paclitaxel (PTX) were manufactured. Using maleimide-thiol chemistry, CPK peptide was then attached to the surface of nanoparticles (CPK-PTX-NPs). MCT1 receptor-overexpressing colorectal cancer (CRC) cells displayed MCT1 receptor-mediated cellular uptake and apoptosis-mediated cell death in CPK-PTX-NPs, but the other nontargeting nanoparticles failed to show MCT1 receptor-mediated cellular uptake. CPK-PTX-NPs were able to decrease angiogenesis in a chick embryo angiogenesis assay by targeting the specific CPK-PTX molecule. PTX-encapsulated and CPK-decorated polymeric nanoparticles, which are selective for the MCT1 receptor and encapsulated and decorated with peptides unique to the CRC, are effective carriers for antineoplastic drug delivery that result in dramatically improved therapeutic efficacy.	(112)
Smith et al; 2020	Solid lipid nanoparticles	Hot and cold homogenization technique	5-Fluorouracil	It was possible to create the SLN-loaded 5-FU through the use of a SMART (Strategic and Unique Method to Advance and Refine the Treatment) for CRC. PEGylated lipids and a mix of surfactants were used to create the SLN. Cytotoxicity, clonogenic assays, flow cytometry, and confocal microscopy were used to investigate the cellular uptake and efficacy of 5FU-SLN in HCT-116 cancer cells. Drug effectiveness investigations on mice with subcutaneous HCT-116 cancer yielded pharmacokinetic (PK) and receptor expression data. 5FU-SLN was the most effective formulation with particle size of 263 ± 3 nm, zeta potential of 0.1 ± 0.02 and entrapment effectiveness of 81 ± 10 percent. -Fu-IC50 SLN value was lower than that of 5-FU (17.7 ± 0.03 µM) by 2.3 times, according to the results of this study. Compared to 5-FU, 5FU-SLN considerably reduced tumour growth, and the area under the plasma concentration-time curve (AUC) of 5FU-SLN was 3.6 times greater than that of 5-FU. Comparing 5-FU-SLN treated animals to 5FU-treated mice, HER2 receptor expression was significantly reduced, but liver and kidney tissues revealed minimal damage at a dose of 20 mg/kg. When compared to 5-FU, 5FU-SLN was highly cytotoxic to HCT-116 cells and greatly reduced tumour growth in mice in the subcutaneous region. An efficient delivery system for anticancer medicines is therefore critical, as evidenced by this finding.	(113)
Serini et al; 2018	Solid lipid nanoparticles	Microemulsion technique	Resveratrol	Resveratrol esterified to stearic acid was used as a lipid matrix for the encapsulation of omega-3 PUFA in the form of solid lipid nanoparticles (SLN). Increase the efficiency of fatty acid incorporation into cells and prevent peroxidation/degradation of these fats was our goal. The antioxidant activity of SLN derived from resveratrol was studied and defined. An increase in the amount of omega-3 PUFAs incorporated into human HT-29 CRC cells and the growth inhibitory effects they had on these cancer cells were seen when the SLN was filled with the fats.	(114)
Kamel et al; 2017	Solid lipid nanoparticles	Ultrasonication method	5-Fluorouracil + (Cinnamon/oregano)	In order to achieve a particle size of 254.77nm, a polydispersity index of 0.28, a zeta potential of +15.26, and an entrapment efficiency percent of 77.3 percent for cinnamon and 69.1 percent for oregano, the experimental model developed suggested an optimal formulation with 2% lipid and 2.3 percent surfactant and 0.4 percent chitosan as the key ingredients. Unencapsulated/encapsulated cinnamon and oregano extracts with or without 5-Flourouracil were tested for cytotoxic activities on HCT-116 cells in phase three. To reduce side effects and allow for continued treatment, this study demonstrated the effectiveness of the 5-Flourouracil combination proposed for treating human colon cancer at low doses.	(115)
Safwat et al; 2016	Gold nanoparticles	Citrate reduction method	5-Fluorouracil	Thioglycolic acid (TGA) and glutathione (GSH) were used to load 5-FU onto gold nanoparticles in order to increase their anticancer activity while reducing their negative effects. Researchers synthesized and evaluated GNPs in different molar ratios of 5-FU/ligand. Colorectal cancer tissue was examined using flow cytometry to determine the anticancer activity of 5-FU/GSH-GNPs. The GNPs were spherical and ranged in diameter from 9 to 17 nm. GNP stability and drug release were studied as a function of salt concentration and solution pH. For both TGA-GNPs and GSH-GNPs, maximal 5-FU loading could be achieved at molar ratios of 1:1 and 2:1. GNPs with a pluronic F127 coating was more resistant to salt. The 5-FU released from GNPs was discovered to be pH-dependent and progressive. In colorectal cancer cells, 5-FU/GSH-GNPs induced apoptosis and halted cell cycle progression. They have a two-fold stronger anticancer effect compared to free 5-FU. These data show that 5-FU's anticancer efficacy can be enhanced by GNPs.	(116)

### Liposomes

A lipid vesicle with a membrane made of amphiphilic phospholipids is called a liposome, and it contains an aqueous volume. All of these compounds are made up of neutral phospholipids like lecithin or cholesterol as well as positively- or negatively-charged ones. Inert, biodegradable and biocompatible liposomes made of phospholipid molecules, which make up most of the membranes in living organisms, are produced by the RES. For the majority of phospholipids, self-assembly in water results in the formation of two or more bilayers or multilayer vesicles with an average bilayer thickness of roughly 5 nm. Liposomes can load medicinal substances selectively by encapsulating hydrophilic drugs in the aqueous core and hydrophobic drugs in the lipid bilayer throughout this process. Oral, intravenous, and rectal administration of liposomes for the treatment of CRC have all been documented in the literature. Panitumumab and cetuximab are two monoclonal antibodies that specifically target ERBB1 (EGF receptor) signaling, which plays a crucial role in the progression and development of colorectal cancer (114). Blood arteries in the vicinity of tumours are the primary target for passive targeting liposomes. Endothelial cell gaps can range from 100 to 780 nm in different cancer types, whereas in normal endothelial cells, the gap is only 5 to 10 nm wide. As a result, liposomes of a size that is acceptable in this context can extravasate into cancerous tissues. Antigens, antibodies and enzymes can all be conjugated to the surface of liposomes in order to boost their potential to target cancer cells more effectively. Proteins overexpressed in tumor cells, such as folate receptors and transferrin receptors, can be targeted by these compounds with a high degree of specificity (EGFRs). Affinity interaction occurs when targeted liposomes arrive at cancer areas, allowing them to aggregate around tumor tissues (115). Shen et al. construct a bifunctional liposome by self-assembly of oxaliplatin-prodrug (Oxa (IV)) conjugated phospholipid and alkylated NLG919 (aNLG), an IDO1 inhibitor, together with other commercial lipids. An NLG919-mediated inhibition of IDO1 in the NLG/Oxa (IV), liposomes can effectively prevent the depletion of tryptophan to immunosuppressive kynurenine in cancer cells, as well as release the cytotoxic oxaliplatin into the cytosol to induce immunogenic cell death (ICD). ANLG/Oxa (IV)-Lip, on the other hand, has been shown to have a extensive blood circulation period, allowing for highly efficient passive tumor homing. Anti-tumor efficacy of such aNLG/Oxa(IV)-Lip is enhanced in both SC and orthotopic CT26 tumours due to significantly primed anti-tumor immunity of enhanced intratumoral CD8+ T cell, cytotoxic cytokines and downregulation of immunosuppressive regulatory T cells, which are present in both tumor types. There’s a lot of potential for future clinical use of this bifunctional NLG/OXA(IV)-Lip due to its good biocompatibility and strong therapeutic performance (116). Alomrani et al. prepared chitosan-coated flexible liposomes (chitosomes) containing 5-FU were developed and characterized to use as a novel approach to target CRC cells. Using film hydration and electrostatic deposition, 5-FU-loaded flexible liposomes, as well as 5-FU-loaded chitosomes, were created. A positive surface charge ranged from 6.1 mV to 14.7 mV for chitosomes, while a negative surface charge ranged from 2.3 mV to 16.3 mV for liposomes, according to the results. Compared to 5-FU solution and liposomes, chitosomes inhibit 5-FU release an *in vitro* drug release investigation. Cytotoxicity tests on the CRC cell line HT-29 revealed that 5-FU-loaded chitosomes outperformed liposomes and the 5-FU solution in killing cancer cells over the long term. It was thus possible to successfully produce chitosomes that carry 5-FU as a nanocarrier in order to potentially harm cells of colorectal cancer (117).

### Polymeric nanoparticles

Due to various characteristics, including size, surface property, and shape, the mononuclear phagocytic system (MPS) quickly opsonizes and clears standard nanoparticle formulations in circulation. These factors are mostly governed by polymer property. Recent research into the effects of polymeric nanoparticle characteristics has proven great therapeutic usefulness in the delivery of medical medicines and bioactive substances (118). EPR effect is a mechanism by which polymeric nanoparticles travel through leaky blood arteries and preferentially aggregate at tumor sites because of their small size and stealth qualities (often between 10nm and 200nm). It is possible to use natural or synthetic polymers in the manufacture of these nanoparticles. Improved medication bioavailability, control of drug release, longer circulation time and reduced non-specific toxicity can be achieved by using polymeric nanoparticles in the medical industry. By boosting the intracellular penetration of medicines into tumor cells, polymeric NPs’ targeting functionality for both active and passive allows them to selectively target certain tissue regions. A regulated and targeted DDS for CRC therapy can take advantage of the biodegradable polymer nanoparticle’s great capabilities (119).

Udompornmongkol et al. developed Curcumin-loaded polymeric NPs for enhanced anti-CRC applications. Curcumin was incorporated into polymeric NPs for increased anti-CRC. Chitosan and gum arabic, two naturally occurring polysaccharides, were used in the emulsification solvent diffusion process to create nanoparticles. Curcumin was found to be encapsulated in carriers with a +48 mV ZP, 136 nm size, and excellent encapsulation efficacy, according to the findings (95 percent). They rectified in their research work that curcumin NPs could withstand hydrolysis by gastric juice or tiny intestinal enzymes, and consequently, it should reach the colon substantially intact, based on an *in vitro* release study. Due to their enhanced cellular absorption, curcumin nanoparticles demonstrated stronger anti-CRC effects than free curcumin. It was so determined that curcumin was successfully encapsulated with superior anti-CRC action in chitosan-gum arabic NPs (120). Badran et al. investigated the activity of 5-FU loaded chitosan coated PLGA NPs (C-5-FU PLGA NPs) and PCL. To deliver cancer treatment, (C-5-FU PCL NPs) were used as carriers. The synthesized NPs had a PDI of 0.30 and had a spherical shape with a particle size range of 188.1–302.2 nm. ZP changed from a negative to a positive value when nanoparticles were coated with chitosan. 5-FU’s entrapment efficiency was found in the range from 32% to 51%. Initially, 5-FU was released rapidly *in vitro*, followed by a steady release profile. CRC cells (HT-29) were significantly inhibited *in vitro* by the C-5-FU PLGA NPs compared to other NPs and medication solution. These findings demonstrate that C-5-FU PLGA NPs are a promising cancer therapeutic delivery vehicle (121). Bhattacharya. S develop chitosan based polymeric NPs of Imatinib (IMT-PNPs) for CRC targeting. Ionic gelation and central composite design were used to make IMT-PNPs. There were 21 batches in which the F10 formulation has been optimized. Approximately, 208 ± 0.01 nm particle size was identified in the improved formulations, as well as a ZP of −32.56 ± 0.03 mV, an in-vitro cumulative drug release of 86.45 ± 0.05%, and a drug entrapment efficacy of 68.52 ± 0.01%. After intravenous delivery of fluorescent nanoparticles, epithelial colon cells display a greater concentration of fluorescent nanoparticles. Because just 0.46 percent of IMT-PNPs formulations had hemolysis as a result of intravenous administration, the formulation is considered safe. Histopathological study of the final formulations found no evidence of tissue injury, indicating that the I.V. mode of administration of the final formulation is safe. The MTT assay shows that entrapped IMT-PNPs cause greater cytotoxicity in CT26 CRC cell lines and it’s this cytotoxicity is better regulated. IMT-PNPs may be a viable method for targeting colorectal cancer utilizing the intravenous route, according to the findings of this study (122).

### Solid lipid nanoparticles

Solid lipid nanoparticles (SLNs) or lipospheres are a promising class of pharmaceutical nanocarriers for regulated drug delivery. Biodegradable and non-toxic lipidic components typically make up SLNs. In addition to being able to transport a range of treatments, SLNs may also carry genetic material (DNA/siRNA), vaccination antigens, and other biomacromolecules. Aqueous colloidal dispersions of solid biodegradable lipids provide the matrix of SLN. Colloid-based carriers, such as the SLN, combine the advantages and prevents the disadvantages of numerous colloidal carriers of its type, such as physical stability, protection from degradation of included labile medicines and regulated release, great tolerability. SLN formulations have been produced and comprehensively studied *in vitro* and *in vivo* for a variety of administration routes (parenteral, oral, cutaneous, ophthalmic, pulmonar, and rectal). To ensure the SLNs’ quality, they must be properly and adequately characterized. Because of the small size of the particles and the dynamic nature of the delivery mechanism, characterizing SLN is extremely difficult (123). Particle size, ZP, lipid modification (polymorphism), degree of crystallinity, and coexistence of additional colloidal structures (miscelles, liposome, super cooled melts, drug nanoparticles), time scale of distribution processes, *in vitro* drug release, surface morphology, and drug content are some of the important parameters evaluated for the SLNs. They may load both hydrophilic and lipophilic medicines, which makes them unique among tiny drug molecules. From popular and convenient modes of administration, such as oral and intravenous administration, the latter ones are quite difficult to provide. SLNs have a lovely interior core structure that can accommodate lipophilic compounds. Being small, these particles have advantages in terms of the biopharmaceutical features of nanoparticle trafficking *in vivo*, followed by drug administration and controlled release at the target site of action. Depending on how they are prepared, they are colloidal in size and can be loaded with hydrophilic and lipophilic medicines. The heated microemulsions from which SLN are made have a versatile component that can be tailored to the kind of medicine and the mode of administration (124). Rajpoot et al. develop and optimize oxaliplatin (OP) loaded SLNs. These SLNs comprise Tween 80, DSPE, Lipoid S75, tristearin, and 1,2-distearoyl-sn-glycero-3-phosphoethanolamine (DSPE). Folic acid (FA) conjugation was made possible by the use of an enhanced SLN formulation developed using the Box–Behnken design. Particle size, ZP, entrapment efficiency (EE), and the shape of the formulations were assessed for several physiological characteristics, such as XRD and DSC. OPSLNs and OPSLNFs with FA-coupled SLNs (OPSLNFs) loaded with OP showed good EE of 49.2 ±  0.38 percent and 43.5 ± 0.59 percent, respectively, and small PS of 146.2 ±  4.4 nm and 158.8 ±  5.6 nm. Results from XRD patterns and DSC analysis showed that OP was evenly distributed in SLNs in an amorphous state. Up to a six-day sustained drug release of OPSLNs and OPSLNFs formulation was demonstrated in an *in vitro* drug release investigation. As compared to OPSLNs and OP solution, OPSLNFs had the strongest anticancer activity on the cell line HT-29. The results of this study show that HT-29 cells are more sensitive to the medication encased in OPSLNFs than OPSLNs and OP solution. As a result, this unique technique may hold promise for the treatment of CRC (125). Senthil et al. evaluated the effectiveness of chitosan-coated-trans-resveratrol (RSV) and ferulic acid (FER) loaded SLNs that conjugated with folic acid (FA) (C-RSV-FER-FA-SLNs) in CRC targeting in relevant models (*in vitro*). A co-encapsulation approach of the stearic acid is used to perform the conjugation of the FAs. Even under acidic conditions, these SLNs show greater durability, demonstrating their potential for use as DDS. Physiochemical evaluations, such as FTIR, XRD, 1HNMR and particle size, ZP and drug release, are also carried out on the optimised formulations. When compared to free RSV-FER, the C-RSV-FER-FA-SLNs efficiently involved and elevated cytotoxicity in cancer cells that resulted in apoptosis, as demonstrated by fluorescence labelling, flow cytometry and western blot analysis. Therefore, it is suggested that this C-RSV-FER-FA-SLNs may be a suitable candidate for new nanodrug formulations in cancer therapy due to its good stability under acidic circumstances (126).

### Gold nanoparticles

When it comes to the ability of AuNPs to serve as an optimum drug carrier and overcome biological obstacles like macrophage clearance, their physicochemical qualities, such as their size, shape, and surface features, are critical. The interaction between membrane receptors and NPs is one of the most essential features that governs the pace of cellular uptake (endocytosis) and hence enhances the accumulation of drug-loaded NPs at the tumor site. In order to avoid early clearance by the MPS organs, the nanoparticle size is crucial. The rate of cellular absorption and accumulation of AuNPs has been described in a number of prior investigations for different AuNP sizes. Tunability of AuNP size during chemical production might thereby enhance efficient delivery of therapeutic agents to selected cells. In addition to traditional methods, scientists are exploring new ways to produce gold NPs, called green synthesis. These systems are known for their safety, environmental friendliness and cost-effectiveness (127, 128). This process is considered non-toxic, environmentally friendly and cost-effective. Green synthesis involves the production of NPs internally and externally using sunlight, electricity and organisms such as fungi, algae and bacteria (129). This technique allows for the production of various types of gold NPs, including nanospheres, nanorods, and nanostars. Lee et al. demonstrated in 2020 that the synthesis of gold NPs heavily relies on green materials such as enzymes, bacteria, plants, and fungi. These advancements in green synthesis offer promising alternatives for the production of gold NPs (130). In a study by Rani et al., the therapeutic effects of biogenic gold nanoparticles derived from Abutilon indicum (AIAuNPs) were investigated in Wistar rats with 1, 2-dimethyl hydrazine (DMH)-induced CRC. The results showed a positive localization of AIAuNPs in colon tumors as assessed by ICP-OES, indicating their bioavailability. Compared with standard paclitaxel, treatment with AIAuNPs increased the level of cellular antioxidant enzymes such as catalase, SOD, GSH, GPx and decreased lipid peroxidation (LPO). In addition, AIAuNPs significantly reduce inflammatory factors (β-catenin and Tcf-4) involved in the Wnt pathway in CRC, while maintaining the expression of apoptotic caspase-9, -8 and -3 and lamin. These findings suggest that AIAuNPs have potential as therapeutic agents for CRC (131).

When nanoparticles interact with lipid bilayer cell membranes, their chemical capabilities, not their size or structure, play a major role. The surface modification of AuNPs is an essential factor in determining their usefulness in drug delivery systems. Oxidative stress and inflammation can result from the overproduction of reactive oxygen species (ROS) in the cells, and MNPs are implicated in both processes (132). ROS has been found to be the primary cause of damage to intracellular compartments such as proteins, DNA, and the cell membrane. An array of intracellular responses, including plasma membrane instability, interference with the anti-oxidant defense system, and cell cycle arrest, as well as genomic damage and interactions with cytoskeleton, proteins and lipids may contribute to cell death. They can harm proteins by binding with their thiol groups, which are linked to oxidation. The cytotoxicity of bio-mediated produced AuNPs was examined in a study on colorectal cancer cells HT-29 and Caco-2. When tested on HT-29 cells, the biogenic AuNPs demonstrated considerable toxicity, but no toxicity on Caco-2 cells. The analysis for apoptotic activity revealed that HT-29 cells had a 13-fold higher percentage of cells in late apoptosis/necrosis than Caco-2 cells, but the percentage of cells in early apoptosis was nearly identical in both cell lines (133). Using two thiol-containing ligands, thioglycolic acid (TGA) and glutathione (GSH), Safwat et al. produced gold NPs to increase 5-FU anticancer activity and reduce its adverse effects. The GNPs were synthesized at various 5-FU/ligand molar ratios and tested utilizing various methods. Flow cytometry was used to examine the anticancer effectiveness of 5-FU/GSH-GNPs in colorectal cancer tissue. The GNPs had a diameter of between 9 and 17 nm and were spherical in form. The effects of salt content and solution pH on GNP stability and drug release were investigated. TGA-GNPs and GSH-GNPs were able to achieve maximum 5-FU loading at a 5-FU/ligand molar ratio of 1:1 and 2:1, respectively. The Pluronic F127 coating on GNPs increased their resistance to salt. A gradual and pH-dependent release of 5-FU from GNPs was observed. 5-FU/GSH-GNPs promoted apoptosis in colorectal cancer cells and halted cell cycle development. Compared to free 5-FU, they demonstrated a two-fold greater anticancer impact. These findings demonstrate that GNPs can improve the antitumor activity of 5-FU (134). The targeted chemo-photothermal treatment of CRC was developed by Emami et al. using doxorubicin (DOX) conjugated with anti-PD-L1 targeting gold NPs (PD-L1-AuNP-DOX). Anti-PD-L1 antibody and DOX have been linked by amide linkage to the terminal end group of lipoic acid polyethylene glycol N-hydroxysuccinimide (LA-PEG-NHS), and PD-L1-AuNP-DOX has been synthesized by attaching a short PEG chain to the surface of AuNP and joining LA-PEG-DOX and LA-PEG-PD-L1. Near-infrared (NIR) irradiation was used to characterize the PD-L1-AuNP-physicochemical DOX’s properties and conduct biological research. An excellent intracellular absorption of DOX was demonstrated in CT-26 cells by the 66.0 percent apoptotic impact of PD-L1-AuNP-DOX (40.0 nm). Apoptosis and cell cycle arrest were increased by PD-L1-AuNP-DOX therapy in combination with NIR irradiation in the *in vitro* proliferation of CT-26 cells. The study shows that synergistic targeted chemo-photothermal therapy in conjunction with PD-L1-AuNP-DOX has a significant promise for treating localized CRC (135).

### Dendrimers

Dendrimers are nanosized macromolecules with tree-like branches and arms originating from a central core (136). Several cationic, neutral, or anionic end groups are present on the arms. Throughout the synthesis process, branches are added to the core at successive levels known as generations. Dendritic macromolecules likely to grow linearly in diameter and adopt a globular shape as dendrimer branches increases (137). Due to their specific physicochemical properties, as well as their biodegradable backbones, dendrimers are suitable for delivering drugs and genes (138–140). Drugs and targeting moieties can be loaded into dendrimer cavities through chemical linkages, hydrogen bonds, or hydrophobic interactions. Multiple dendrimers have been investigated for cancer therapeutics, including polyamidoamine (PAMAM), polypropylene imine (PPI), poly(ethylene glycol) (PEG), Bis-MPA (2,2-bis(hydroxymethyl) propionic acid) and 5-ALA (5-aminolevulinic acid) (141). Dendrimer-DOX was studied by Mignani et al. which showed that it was 10 times less harmful than free DOX after being exposed to C-26 CRC cells for 72 hours. When BALB/c mice with C-26 CRC tumors were given dendrimer-DOX, the tumor uptake was 9 times greater than with free DOX at 48 hours and had a half-life of 16-hour. The mice survived for two months with a single injection of dendrimer–DOX (141).

In a research conducted by Zhuo et al., different generations (0.5-5.5) of 5-FU-dendrimer conjugates were synthesized, demonstrating enhanced controlled release properties for the anticancer drug 5-FU (142). Moreover, the conjugation of DOX with PEGylated dendrimers resulted in improved circulation time, decreased drug accumulation, and reduced toxicity. When administered subcutaneously in a mice with highly invasive CRC C26 cells, these dendrimer-conjugated formulations showed the ability to overcome the known resistance of these tumor cells to doxorubicin (143). Additionally, dendrimers have shown potential in preventing the initiation of metastasis by selectively binding to and cytotoxically eliminating circulating tumor cells (CTCs) (144). Due to these promising attributes, dendrimers are often referred to as “therapeutic dendrimers” and warrant further investigation and attention in the field of cancer-targeted therapy (145).

### Quantum dots

Quantum dots (QDs) are tiny semiconductor nanoparticles with a diameter smaller than 10 nm. Due to their small size, they are often used as fluorescent labels in medical imaging or incorporated into nanostructure scaffolds for diagnosis and treatment purposes. QDs have been extensively studied in theoretical quantum mechanics, and their optical properties, which depend on their size and composition, make them valuable in medical imaging, especially for the gastrointestinal tract. For instance, in CRC, ODs labelled with bevacizumab, an antibody that targets VEGF, have shown promise in non-invasively tracking the overexpression of VEGF. These theranostic QDs not only possess therapeutic capabilities but also enable the visualization of antibody binding specificity (146). Additionally, a patented approach involves the use of porphyrin carbon QDs conjugated with cetuximab (C225-PCQD) for imaging and photodynamic therapy. These QDs have the ability to accumulate in CRC cells that have elevated levels of EGFR (147).

### Polymeric micelles

Polymeric micelles (PMs) are self-assembled structures formed by amphiphilic block copolymers in water-based solutions. These micelles possess a hydrophobic core and a hydrophilic shell, making them suitable for encapsulating hydrophobic drugs and improving their solubility (148). PM-based carriers can be easily developed and can be optimized for drug delivery. Additionally, they can be functionalized with targeting ligands to enhance their accumulation at tumor sites, reduce side effects, and enable controlled release of drugs over an extended period (149, 150). Recent study has focused on the development of pH-responsive copolymers for optimized delivery of anticancer drugs in colon cancer treatment. These micelles exhibit pH sensitivity and effectively target colon tissues, achieving controlled drug release rates of over 80% (151). Hence, they are regarded as “smart” nanocarriers for delivering anticancer drugs and imaging agents, with potential applications in therapeutics and diagnostics. Notably, several PM formulations loaded with drugs have entered clinical trials for cancer treatment. For instance, Genexol^®^-PM, a PM formulation loaded with paclitaxel (PCX), is undergoing phase IV clinical trials for CRC, and other trials aim to explore its efficacy in ovarian, lung, cervical, and pancreatic cancers. Preclinical studies on multidimensional PMs are also underway, highlighting their potential as promising platforms for drug delivery and cancer therapy, deserving further investigation and attention (152).

### Mesoporous silica nanoparticles

Mesoporous silica nanoparticles (MSNs) are a class of materials composed of silica (SiO2) that have attracted significant attention in drug delivery due to their unique porous structure, capable of accommodating large amounts of bioactive molecules. MSNs offer adjustable cavity sizes within the range of 50-300 nm, lower toxicity, easy uptake by cells, and resistance to heat and variable pH conditions (153). A hybrid system called MSN-protamine (MSN-PRM) has been developed to enable selective drug release in cancer cells, which can be activated by specific enzymes to initiate anticancer activity (154). By conjugating MSNs with hyaluronic acid, the loading capacity of doxorubicin (DOX) into the MSNs is significantly increased compared to unmodified MSNs. This improvement resulted in improved cellular uptake and cytotoxicity against human cancer cells. In addition, functionalization of MSNs with polyethyleneimine-polyethylene glycol (PEI-PEG) or PEG increased epirubicin hydrochloride (EPI) loading and improved its antitumor activity (155). Silica nanoparticles have been used in the treatment of CRC when combined with photons to selectively destroy CRC cells (156). Silica-based nanoshells encapsulate photosensitizing molecules, facilitating their uptake by tumor cells. When exposed to light, the photosensitive device releases oxygen molecules, effectively killing cancer cells (157). Clinical trials are currently investigating this technology in cancer treatment. Additionally, nanoparticles have shown promise in molecular imaging of cancer cells, enabling earlier diagnosis and targeted DDS.

## Magnetic and metallic nanoparticles as photosensitizers

Metallic and magnetic nanomaterials possess distinct magnetic, optical, and photothermal properties that make them valuable in various biomedical applications. Among these materials, iron oxide stands out as a notable metallic nanomaterial with versatile uses. Its exceptional magnetic properties enable its application in imaging techniques and targeted drug delivery (158). Metallic NPs can be combined with other nanomaterials and integrated with therapies like photothermal therapy (PTT). Iron oxide nanoparticles, specifically, exhibit excellent biodegradability within the human body, as the iron ions can be naturally adjusted. Recent research suggests that smart multifunctional magnetic nanovesicles containing the antibody-targeting peptide AP1 (MPVA AP1) hold promise as effective anticancer agents (158). These nanovesicles demonstrate remarkable selectivity and targeting towards CRC cells while ensuring minimal drug leakage without magnetic field stimulation. Additionally, nanovesicles loaded with doxorubicin release the drug rapidly, accurately, and under precise control when exposed to a high-frequency magnetic field. Consequently, smart magnetic nanovesicles like MPVA-AP1 exhibit significant potential for delivering specific doses and achieving sustained drug release in antitumor applications. Iron oxide nanoparticles also increased hyperthermia effects and prove highly beneficial in CRC diagnosis. For instance, PLGA NPs loaded with 5-FU and iron oxide induce greater DNA damage in HT-29 colon tumor cells compared to hyperthermia alone (159). Other studies reveal the controlled release of PCX and super-paramagnetic iron oxide (SPIO) from PEAL Ca micelles, with release rates influenced by pH levels. Cell culture experiments further demonstrate successful absorption of PTX-SPIO-PEALCa by CRCLoVo cells, while PCX is internalized by lysosomal cells, effectively inhibiting CRC LoVo cell growth. Thus, micelles offer substantially potential as well as greater drug release methods for CRC treatment using MRI imaging (160).

## Carbon-based nanoparticles

The carbon-based nanomaterial family encompasses various members such as fullerenes, carbon nanotubes, graphene, nanodiamonds, and carbon-based quantum dots (161). These nanomaterials exhibit exceptional physical and chemical properties, including mechanical strength, electrical conductivity, thermal stability, optical characteristics, and chemical reactivity. As a result, they have attracted significant attention and are being extensively researched for a wide range of applications, particularly in biomedicine. They hold promise as carriers for therapeutic agents in disease treatment, tissue regeneration, and cell and tissue imaging. Furthermore, their anti-bacterial and anti-inflammatory activity are also being extensively investigated (162).

Carbon nanotubes (CNTs) are a widely studied type of carbon nanoparticles in the field of biomedicine. These are cylindrical structures made of extruded graphene sheets with a diameter of less than 1 µm and a nanoscale length (162). Their large surface area, needle-like structure, high thermal conductivity, and chemical stability make them suitable for various applications, including immunotherapy, diagnostics, gene therapy, and as carriers in DDS (163). Many strategies have been developed to use CNTs as anti-inflammatory agents. For example, single-walled carbon nanotubes (SWCNTs) conjugated with a synthetic polyampholyte have demonstrated enhanced anticancer effects of paclitaxel in Caco-2 and HT-29 cells compared to paclitaxel alone (164). Similarly, Eudragit^®^-irinotecan-loaded CNTs have shown improved efficacy in cancer treatment (165). Moreover, infrared light-activated oxaliplatin and mitomycin C-coated CNTs exhibited higher drug delivery and localization in colon cancer cell lines (166). SWCNTs modified with TRAIL, a ligand that induces apoptosis in cancer cells, have demonstrated significantly increased cell death compared to TRAIL delivery alone in carcinoma cell lines (167).

## Cyclodextrin complexes

Cyclodextrins (CDs) are cyclic oligosaccharides composed of glucose units linked by glycosidic bonds. They come in three forms: α-CD, β-CD, and γ-CD, each consisting of six, seven, or eight glucose units, respectively (168, 169). The unique structure of CDs resembles a hollow truncated cone with a hydrophobic cavity and a hydrophilic outer surface, thanks to the chair arrangement of the glucopyranose groups. This structure enables the encapsulation of hydrophobic drugs within the CD cavity, forming host-guest complexes without requiring complex chemical reactions (170, 171). Furthermore, CDs can form reversible inclusion complexes with various guest molecules, allowing for both drug loading and controlled drug release at specific sites as needed (172).

In a recent study by Bai et al., a modified γ-CD containing mannose was utilized to deliver regorafenib and effectively target colorectal cancer cells (173). The modified CD formed various types of channels that specifically targeted cancer cells, leading to cell death. The study demonstrated sustained release of the drug, resulting in increased apoptosis and a reduction in tumor supportive factors and pro-inflammatory cytokines. This research highlights the potential of CDs in targeting cancer cells, which can be further enhanced by incorporating appropriate targeting agents. Another study focused on using a polycationic β-CD complexed with camptothecin (CPT), a potent drug, to enhance its stability for the treatment of early- and late-stage colon cancers (174).

Accurately distinguishing cancerous cells from normal tissues is crucial for effective cancer diagnosis. Nanoengineering offers a promising solution by enhancing the targeting and luminescent properties of various materials, enabling their use in biomedical applications. This advancement has led to the development of bioimaging techniques that utilize nanomaterials for the identification of different types of tumors. For instance, Mortezazadeh et al. developed a targeted nanocontrast agent for magnetic resonance imaging (MRI) using gadolinium (Gd) nanoparticles coated with a β-CD-based polyester and folic acid (FA). This nanoparticulate contrasting agent enables precise localization and improved tissue discrimination, enhancing the accuracy of cancer diagnosis. The polymer coating not only provides stability to the nanoparticles in biological conditions but also prevents leakage into normal tissues. The coated spherical Gd nanoparticles, with a diameter ranging from 75 to 95 nm, demonstrated non-toxicity towards normal human breast cells (MCF-10A) in MTT assays, unlike free Gd2O3 (175). Although nanoscale drug delivery vehicles have made significant progress, further advancements are necessary to meet the clinical standards of care. CDs hold great promise as versatile agents capable of fulfilling multiple roles at the nanoscale level.

## Nanoimmunotherapeutics and nanovaccines

Nanoimmunotherapeutics has emerged as a promising approach for treating CRC by combining nanotechnology and immunotherapy. Zhang et al. developed NPs capable of delivering immune checkpoint inhibitors, such as anti-PD-1 or anti-CTLA-4 antibodies, directly to the tumor microenvironment. This targeted delivery enhances immune cell activation and overcomes mechanisms of immune evasion (176). Additionally, Nanoimmunotherapeutics can incorporate immune stimulants like Toll-like receptor agonists to further boost immune cell activity against tumors (177). Preclinical studies in CRC models have demonstrated the effectiveness of Nanoimmunotherapeutics, showing improved tumor regression, prolonged survival, and enhanced immune responses (177).

Nanovaccines have emerged as a promising approach for treating CRC by utilizing the immune system to target and eradicate cancer cells. These nanoscale vaccines are specifically designed to deliver tumor-specific antigens, adjuvants, and immunomodulators, thereby eliciting a potent and protective immune response against tumors. The nanovaccine uses the unique properties of nanoparticles to improve antigen presentation, activate the immune system and boost immunity against cancer (178).

A nanovaccine that comprises certain tumor antigens like carcinoembryonic antigen (CEA) or mucin 1 (MUC1) that demonstrates a response resistance to sickness and tumor progression has demonstrated promising outcomes in clinical models. Additionally, Toll-like receptor agonists or NPs containing internal components may be used in nanovaccines and nanoimmunotherapies to enhance immunity and antigen presentation (179). Although clinical studies and the development of nanovaccine and nanoimmunotherapy for CRC are still in their early phases, they have the potential to enhance the immune system’s performance in CRC patients (180).

## Clinical trials for nanotechnology used in CRC

Despite the fact that several nano formulations are undergoing clinical studies, there aren’t many of them being utilized to treat CRC. **Table 4** provides a summary of some of the nanoformulations employed for the suitable clinical studies against the CRC. In a research study, a smooth-thermosensitive liposomes containing doxorubicin called Thermodox^®^ is intended for usage in combination with thermal ablation. Thermodox^®^ in combination with thermal ablation was investigated for safety, viability, and effectiveness in treating liver metastases in CRC in an open phase II investigations (NCT02181075) (181). In contrast to targeting NPs, while passive targeting NPs have already received FDA approval as cancer nanotherapeutics, active targeting NPs are still in the early phases of clinical trials (182).

**Table 4:** Most recent clinical trials with nano formulations for CRC therapy.

Nanocarrier Used	Drug (Active Agent)	Applications	Clinical Trial Status	Sources
Regulatory lymphocytes (Tregs): anti-CTLA-4 ipilimumab and anti-PD-L1 atezolizuma	Cytotoxic antibodies expressed on surface of Tregs	Colorectal cancer	FDA approved	(138)
Polymeric NPs + cetuximab + somatostatin analogue	Combination of NPs Somatostatin and Cetuximab analogue	Metastatic colorectal cancer	Phase I trial	(139)
NKTR-102/IRI	Formulation for prolonged release of IRI conjugated with PEG/IRI	Metastatic CRC with KRAS-mutant	II clinical trial	(139)
PEG-PGA polymeric micelle	SN-38	Colorectal, lung, & ovarian cancers	Phase II trial	(140)
Liposome	Doxorubicin	Colon cancer with liver metastasis	Phase II trial	(141)
CPX-1 liposome	Liposomal IRI (irinotecan) hydrochloride and floxuridine	Advanced colorectal cancer	Phase II trial	(139)
Cyclodextrin nanoparticle	Camptothecin	Solid tumors, rectal cancer, renal cell carcinoma, non-small cell lung cancers	Phase I/II trial	(142)
Carbon NPs	Carbon NPs	Laparoscopic surgery of colorectal cancer	Phase I trial	(139)
TKM-080301	Lipid NPs with serine/threonine kinase inhibitor	Colorectal cancer with liver metastases and ovarian, gastric, esophageal, and breast cancer	Phase I trial	(139)
Nal-IRI	Liposomal IRI	Colorectal cancer along with advanced gastrointestinal cancers	Phase I/II trial	(139)
PEP02 liposome	Liposomal IRI hydrochloride + 5-FU and LV (leucovorin)	Metastatic colorectal cancer	Phase II trial	(139)
PEG-rhG-CSF	PEGylated recombinant human granulocyte colony stimulating factor (CSF)	Solid malignant tumors (colorectal, ovarian, lung, head, and neck cancer)	Phase IV trial	(139)
PROMITIL	PEGylated liposomal mitomycin C	Metastatic colorectal cancer	Phase I trial	(139)
Silica NPs	Fluorescent cRGDY-PEG-Cy5.5-C dots	Colorectal malignancies	Phase I-II trial	(139)
MM-398	Liposomal IRI	Advanced cancer of unresectable nature	Phase Ib trial	(139)

Furthermore, it has been seen in clinical studies that nanoplatforms typically decrease the toxicity of drugs rather than increase their efficacy. The majority of NPs, including actively targeted nano preparations, accumulate at tumours based on EPR effect, but this effect is more persistent in animals, whereas there are variations in the EPR effect for CRC patients, which will affect the efficacy of nano preparations (183, 184).

## Patents approved for nanotechnology in the treatment of CRC

A few of the clinical trial-validated nanomaterials have been trademarked for commercial use in given in **Table 5**. Theragnostic formulations are new approaches to CRC treatment that try to forecast the results of a specific treatment, for example, by identifying individuals who will respond to a drugs more favourably or by giving information on how a drug is acting (185). For example, Wu et al. developed porphyrin carbon QDs coupled with cetuximab (C225-PCQD), which have been patented as an imaging and photodynamic treatment strategy due to their ability to aggregate in colon cancer cells that overexpress the EGFR receptor (186). Also, a liposomal IRI formulation with 5-FU, LV, and an EGFR inhibitor has also been granted patent protection by Merrimack Pharmaceuticals for the treatment of metastatic CRC with a wild-type RAS mutation (187).

**Table 5:** Recent patents related to nanoformulations for the management and treatment of CRC.

Nanocarrier/ Nanoparticles	Details of patent	Molecule	Year of Patent Granted	Patent Number	Sources
Gold metallic NPs	Fluorouracil (5-FU) INCORPORATED IN Metallic NPs for connecting polynucleotide	Anticancer drug with a pyrimidine group or a purine group such as 5-FU	2014	US8673358B2	(146)
Liposome	Liposomal IRI + 5-FU/LV(leucovorin) and an EGFR inhibitor	Irinotecan(IRI)	2017	WO2017172678	(147)
PEG-modified cationic liposome	shRNA against TS(thymidylate synthase) attached to cationic modified liposome with PEG	shRNA	2014	ES2653923	(148)
Quantum points of porphyrinic carbon	Quantum point of porphyrin carbon conjugated with cetuximab biocompatible	Cetuximab	2018	US20180125976A1	(144)
Glyceryl mono fatty acid ester	NPs of glyceryl monofatty acid ester, chitosan, and therapeutic agent	Antineoplasic agent	2012	US8242165B2	(149)

## Conclusion and future perspectives

A primary goal of the center for disease control and prevention is to prevent cancer, diagnose it early, improve the health of those who have it, and decrease the financial burden of treating it. Following its tremendous success, there are some crucial elements that need further examination. In the typical bench-top technique, it has been difficult to control the nanoparticle size, resulting in batch-to-batch variance. In order to attain more atomization and large-scale capability, additional design work is needed. Nanoparticles with desired particle sizes and distributions can be manufactured continuously in a reproducible, large-scale way using this approach. Nanoparticles also tend to congregate in production and physiological settings. This physical instability can be lessened by mixing in a little amount of NaCl to the gelation media, but there are still other possibilities to consider. Innovative ionic gelation techniques may lead to nanoparticles that are stronger and more stable. Nanoparticles made from chitosan offer a promising start in this direction. In addition, further research is needed to develop stable and effective nanoparticle-based powder compositions. In the treatment of CRC, CRC-targeted nano-DDS can alter the distribution and release of drug in people by accumulating in CRC, which improves therapeutic efficacy and lowers adverse effects compared to conventional therapy approaches. Overall, the results have demonstrated that synthesised gold NPs may be effective anti-colon cancer medications because of their distinctive optical features, which make them valuable in imaging and photothermal treatment. These nanoparticles can also be altered using specific ligands to allow for specific distribution to tumor locations. For the treatment of CRC, researchers have looked at the use of gold nanoparticles to increase the results of radiotherapy and photothermal therapy (188).

The treatment approaches of CRC may change in the future. Using nanoparticles with ligands or antibodies on their surface to deliver substances specifically to tumor cells is one such strategy. Exploiting the increased EPR effect is another strategy that takes advantage of tumours’ aberrant blood arteries and impaired lymphatic drainage. This improves drug delivery by allowing nanoparticles to collect preferentially in tumor tissue. Nanomaterials can also be developed to react to certain stimuli present in the tumor microenvironment, such as pH, temperature, or enzyme activity. This enhances the efficiency of therapy by enabling regulated drug release at the tumor location. Additionally, imaging capabilities can be added to nanomaterials, enabling non-invasive monitoring of medication distribution, tumor targeting, and therapeutic response. These developments could help assist personalised medicine and enhance CRC treatment plans. Furthermore, nanomaterials can be engineered with imaging capabilities, allowing for non-invasive monitoring of drug distribution, tumor targeting, and treatment response. These advancements have the potential to support personalized medicine and optimize treatment strategies for CRC.

This review summarizes and classifies the colon-targeted NPs from the perspective of targeting power, showcasing the diversity and innovation of NPs targeting CRC in academic research. Ultimately, more preclinical and clinical testing is needed to bring gold nanoparticles to the market. There are now several clinical trials being conducted on the use of NPs in CRC. However, a number of clinical phases still need to be completed by the majority of these approaches before they can be commercialized. Toxicology, bioavailability, side effects, cost-effectiveness, and biocompatibility need to be studied further in preclinical and clinical settings. The literature that is now accessible and the research that is being done on the use of NPs in the treatment of cancer clearly suggest that treatments utilizing nanoformulations can simultaneously be used for diagnostic and therapy based on their functionalization and contents are especially promising.

Additionally, new patents for DDS based on nanotechnology are being explored. To ascertain their applicability, adverse effects, removal procedures, and therapeutic benefits, clinical studies are being conducted on them. There has been a rise in medical device and medication delivery system research as a result of the advancements in nanotechnologies. It was possible to create multipurpose platforms, like nano theranostics, using nanotechnology to create medical goods with several modes of action. As a cancer therapy, preclinical studies with nanomaterials have demonstrated their efficacy. Research into immunotoxicity testing, nanoparticle surfaces, and drug fraction encapsulation and decapsulation has shown the importance of nanotechnology in the field of medicine in the 21st century. In biomedical research, the development of DDS with the potential to alter tissue absorption, biodistribution of drugs, and pharmacokinetics of therapeutic agents is critical. Current medical research is focused on nanoparticles. When it comes to developing new treatments for diseases, researchers have focused on using nanotechnology. Anti-degradation, as well as targeted and controlled release, are all possible with medicinal molecules combined with nanocarriers. Numerous nanocarriers for cancer treatment and diagnosis have been developed during the past 20 years as a result of rapid advancements in nanoscience, technology, and industry and cancer pathology (138). However, only few numbers of nano-drugs have been successfully produced and involved in clinical settings.

## Author contributions

AJ: Investigation, conceptualization, writing original draft. SB: Validation, designing, methodology, reviewing and editing. The authors declare that the work was done, analysed, drafted by all the authors of this manuscript. All the authors had read and approved the final manuscript. All authors contributed to the article and approved the submitted version.
